# The Geometry of Circulatory Shock: A Conceptual Multi-Scale Lagrangian Framework for Physiology-Informed Hemodynamic Phenotyping

**DOI:** 10.3390/jcm15114283

**Published:** 2026-06-01

**Authors:** Athanasios Chalkias, Konstantina Katsifa, Stavroula Amanetopoulou, Georgios Karapiperis, Antonios Destounis, Ioanna Iatrelli, Eleni Laou, Athanasios Prekates, Paraskevi Tselioti

**Affiliations:** 1 Institute for Translational Medicine and Therapeutics, University of Pennsylvania Perelman School of Medicine, Philadelphia, PA 19104-5158, USA; 2 Outcomes Research Consortium^®^, Houston, TX 77030, USA; 3 Department of Critical Care Medicine, Tzaneio General Hospital, 18536 Piraeus, Greece; nkatsifa@gmail.com (K.K.); amanetopoulou@yahoo.com (S.A.); dr.giorgio@yahoo.gr (G.K.); antdestounis@gmail.com (A.D.); prekatesa@yahoo.com (A.P.); ptselioti@hotmail.com (P.T.); 4 Department of Anesthesiology, Tzaneio General Hospital, 18536 Piraeus, Greece; iatrellioanna@gmail.com; 5 Department of Anesthesiology, Agia Sophia Children’s Hospital, 11527 Athens, Greece; elenilaou1@gmail.com

**Keywords:** hemodynamics, computational modeling, inverse modeling, topological data analysis, precision phenotyping, critical care, decision support

## Abstract

Background: Hemodynamic failure remains a major determinant of mortality in critical illness, yet its detection is often delayed because conventional monitoring relies predominantly on Eulerian measurements that quantify pressure and flow magnitude without resolving the spatial and temporal organization of circulation. Consequently, clinically significant states of dysfunction may persist despite apparently stable hemodynamic indices. The Geometry of Shock is a conceptual and hypothesis-generating multi-scale framework intended to integrate established cardiovascular physiology with emerging computational approaches for the analysis of circulatory dysfunction. Framework: The proposed framework combines Guytonian venous return physiology and cardiopulmonary interactions with Lagrangian flow topology, geometric representations of circulatory equilibrium, topological data analysis, and physics-constrained inverse modeling. Rather than focusing exclusively on static thresholds of pressure and flow, the framework proposes a structural interpretation of circulation centered on the dynamic organization and coherence of blood transport across cardiovascular domains. Within this paradigm, under-recognized hemodynamic phenotypes—including stressed volume failure, oscillatory shock during spontaneous breathing, macro–microcirculatory decoupling, and pulmonary vascular pressure–flow dissociation—may emerge from disrupted coupling between vascular, cardiac, pulmonary, and microcirculatory systems. These states may represent reversible structural transitions in venous return geometry and cardiopulmonary interaction preceding overt circulatory collapse. Conclusions: By reframing shock as a disorder of circulatory structure and coherence rather than solely a deficit in flow, this framework proposes a mechanistic foundation that may support future approaches aimed at earlier recognition of instability, improved physiological characterization of hemodynamic phenotypes, and future development and prospective validation of physiology-informed computational decision-support strategies in critical care. These concepts remain exploratory and hypothesis-generating rather than clinically validated.

## 1. Introduction

Hemodynamic failure remains a fundamental determinant of mortality in critical illness, regardless of underlying cause. Despite substantial advances in monitoring technology, clinical interpretation of circulatory status continues to rely predominantly on Eulerian measurement paradigms, in which pressure and flow are assessed at fixed spatial locations [1]. Contemporary platforms generate high-resolution time-series data, enabling analysis of waveform morphology, pulse pressure variation (PPV), and related dynamic indices. However, these variables remain fundamentally local descriptors of magnitude rather than structure. They quantify how much flow occurs at a point, but not how flow is organized across space and time. Consequently, current monitoring frameworks lack spatial integration and cannot reconstruct the material history of blood transport or quantify distributed energy dissipation across the vascular network [2]. This limitation contributes to well-recognized clinical paradoxes in which mean arterial pressure (MAP) appears normalized while tissue hypoperfusion and metabolic failure persist, reflecting undetected topological breakdown within the circulatory system [3].

The cardiovascular system is not a collection of rigid conduits but a nonlinear, multiscale dynamical network whose global behavior emerges from the coupled interactions of cardiac function, venous return, vascular compliance, pulmonary mechanics, autonomic regulation, and microcirculatory recruitment. In critical illness, perturbations in this coupled system can induce regime shifts that are mathematically predictable yet clinically under-recognized. States such as occult hypoperfusion represent failures of structural coherence between macro- and microcirculatory domains, in which adequate global driving pressures fail to translate into effective tissue-level perfusion [4,5]. These phenomena cannot be resolved through threshold-based monitoring alone because they reflect breakdown in system organization rather than isolated deviations in scalar variables.

In this article, we propose a physiology-informed conceptual framework that integrates established Guytonian venous return physiology with emerging computational methodologies, including multiscale modeling, topological flow analysis, and physics-constrained inverse approaches. By combining structural descriptors of flow organization with mechanistic cardiovascular principles, the framework is intended to support probabilistic characterization of complex or under-recognized hemodynamic phenotypes using sparse bedside data, while bridging first-principles fluid dynamics with interpretable, physiology-constrained computational decision-support strategies. Importantly, the present manuscript does not describe a prospectively validated computational platform or clinically deployed diagnostic system. Rather, its objective is to outline a translational roadmap for the future development, refinement, and prospective validation of interpretable computational hemodynamic phenotyping approaches for physiology-informed resuscitation.

## 2. The Four Under-Recognized Hemodynamic Phenotypes: A Mechanistic Approach

The multi-scale framework presented here moves beyond classifying shock by flow, pressure, or oxygen delivery alone. Instead, it may help identify structural and phase-domain failures that reflect a fundamental breakdown of circulatory efficiency. These phenotypes are described as “under-recognized” not because they are necessarily rare in prevalence, but because they are frequently under-detected or misclassified within conventional pressure- and flow-based monitoring paradigms. Hemodynamic incoherence, for example, describes a state of macro–microcirculatory decoupling in which microvascular dysfunction persists despite apparent restoration of macrocirculatory variables, highlighting why normalized MAP or cardiac output (CO) may not guarantee adequate tissue perfusion [4,5]. This phenomenon illustrates a clinically important form of an under-recognized circulatory failure in which conventional systemic hemodynamic targets may falsely suggest successful resuscitation despite persistent tissue hypoxia and impaired capillary flow. Similarly, occult hypoperfusion and stressed volume (Vs) failure may occur despite apparently stable vital signs or preserved total blood volume, particularly when reductions in Vs and venous return gradients are not captured by routine bedside monitoring [6,7,8,9,10]. These states may therefore remain misclassified as simple hypotension or fluid-responsive hypovolemia despite fundamentally different underlying physiology. Oscillatory shock during spontaneous breathing represents another under-recognized phenotype in which exaggerated cardiopulmonary interactions and large negative pleural pressure swings induce cyclic RV loading instability and respiratory-phase hemodynamic collapse that may be mistaken for conventional preload responsiveness [11,12,13]. Right ventricular dysfunction, acute right-heart failure, and pulmonary vascular pressure–flow dissociation are likewise diagnostically challenging in critical illness and ARDS, where RV–pulmonary vascular coupling may deteriorate without being adequately reflected by standard systemic hemodynamic indices, often requiring advanced echocardiographic assessment for recognition [8]. Together, these observations support the concept that clinically important hemodynamic phenotypes may remain under-detected or misclassified within conventional ICU monitoring paradigms focused predominantly on static pressure and flow measurements. Table 1 summarizes the four key, under-recognized hemodynamic phenotypes that our approach is designed to uncover.

Stressed volume (Vs) failure is characterized by functional hypovolemia. The core mechanism is a dramatic increase in systemic venous capacitance (Csys), often due to impaired sympathetic tone or chronic disease (e.g., diabetes-induced autonomic dysfunction). This leads to a profound decrease in the Vs, causing the mean circulatory filling pressure (Pmcf) to collapse toward the right atrial pressure (RAP) and thus abolishing the venous return gradient. The clinical presentation includes hypotension and low central venous pressure (CVP), mimicking true hypovolemia, yet the response to fluid is minimal or detrimental, highlighting the need for -adrenergic vasoconstriction or arginine vasopressin (AVP) to restore capacitance.

Oscillatory shock during spontaneous breathing involves profound heart-lung interaction failure. Large, negative pleural pressure swings generated during intense spontaneous breathing (e.g., during a spontaneous breathing trial—SBT) dramatically increase right ventricular (RV) afterload [11,12]. This causes cyclic, beat-to-beat instability of RV stroke volume, leading to a phase-locked respiratory instability of systemic output (pulsus paradoxus or large PPV) [12,13]. Therapy must focus on reducing inspiratory effort and RV afterload sensitivity.

In patients with hemodynamic incoherence (macro-microcirculatory decoupling), a common but under-recognized state, global indices [MAP, CO] are normalized or seemingly adequate, yet the microcirculation remains critically impaired due to microvascular derecruitment and glycocalyx injury [14]. This results in persistent cellular dysoxia, indicated by signs like mottling, high lactate, and low capillary refill, despite misleading “normal” macro-hemodynamics [15]. Management shifts from optimizing global flow to restoring microvascular pressure and coherence.

Pulmonary vascular pressure–flow dissociation is an RV-pulmonary arterial coupling failure [16], where the pulmonary vascular resistance (PVR) demonstrates a non-linear or paradoxical response to small changes in lung volumes [e.g., positive end-expiratory pressure (PEEP) titration or position change]. Abrupt loading shifts cause acute dilation and systemic hypotension, indicating a loss of control over the right heart’s operating point [17].

## 3. Modern Spatial and Temporal Hemodynamic Analysis

Traditional hemodynamic monitoring relies primarily on Eulerian measurements that describe pressure and flow magnitude at isolated points in time. Although these variables remain clinically essential, they provide limited information regarding the spatial and temporal organization of circulation, the stability of flow transport, or the dynamic coupling among cardiovascular compartments. Consequently, important transitions toward circulatory instability may remain undetected until overt hemodynamic collapse occurs.

Recent advances in dynamical systems analysis, Lagrangian flow topology, and topology-informed computational physiology provide an opportunity to reinterpret shock as a disorder of circulatory structure and coherence rather than solely a deficit in pressure or flow. Within this framework, perturbations in venous return, ventricular interaction, pulmonary vascular coupling, and microcirculatory organization can be conceptualized as structural transitions within a multiscale hemodynamic system. This perspective requires integration of advanced mathematical tools derived from chaos theory, transport topology, inverse modeling, and physiology-constrained computational inference [18]. These include Lagrangian coherent structures (LCS), finite-time Lyapunov exponent (FTLE) analysis, and topological data analysis (TDA).

**Figure 1:**
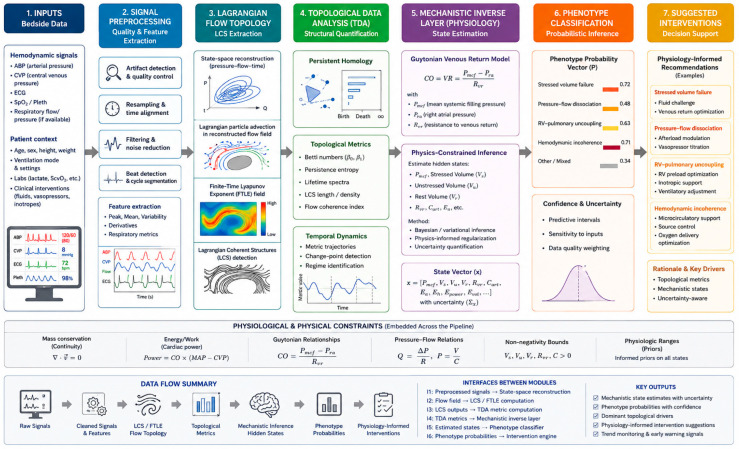
Framework architecture and computational data flow for topology-based precision hemodynamic phenotyping. The figure illustrates the proposed multiscale computational workflow integrating bedside physiological monitoring with topology-informed mechanistic inference. Continuous and intermittent clinical inputs, including arterial pressure, central venous pressure, electrocardiography, respiratory signals, echocardiographic parameters, laboratory measurements, and therapeutic interventions, undergo signal preprocessing, quality control, temporal alignment, and feature extraction. Processed waveforms are subsequently analyzed using Lagrangian flow topology methods, including state-space reconstruction, finite-time Lyapunov exponent mapping, and Lagrangian coherent structure (LCS) extraction, followed by topological data analysis (TDA) to quantify structural coherence, persistence metrics, and temporal dynamical transitions. These outputs are integrated within a physiology-constrained inverse modeling layer based on Guytonian venous return principles and cardiopulmonary interaction physiology to estimate latent hemodynamic states, including stressed, unstressed, and exploratory rest volume-related conditions, venous return gradients, vascular resistance, and circulatory efficiency metrics. The inferred mechanistic state vector is subsequently mapped to probabilistic classification of under-recognized hemodynamic phenotypes, including stressed volume failure, oscillatory shock, macro–microcirculatory decoupling, and pulmonary vascular pressure–flow dissociation, together with uncertainty estimates and physiology-informed therapeutic considerations. Physiological conservation laws and pressure–flow relationships are embedded throughout the computational pipeline as mechanistic constraints. The figure is conceptual and intended to illustrate the proposed translational framework rather than a prospectively validated clinical platform.

The proposed computational architecture is intended to integrate bedside physiological monitoring with topology-informed mechanistic inference. A structured workflow summarizing the proposed data flow architecture, signal preprocessing steps, topological feature extraction, mechanistic inverse modeling, phenotype classification, and physiology-informed decision-support strategy is presented in Figure 1 and described in detail in the Supplemental File.

### 3.1. Lagrangian Coherent Structures and Flow Topology

**Figure 2:**
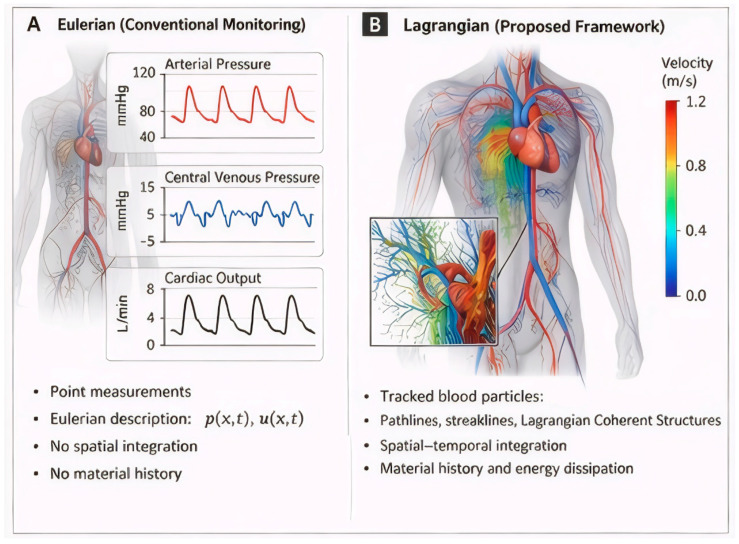
From Eulerian to Lagrangian hemodynamics: conceptual transition from point-based monitoring to flow topology analysis. Panel (**A**) illustrates the conventional Eulerian paradigm of hemodynamic monitoring, in which pressure and flow are measured at fixed spatial locations (e.g., arterial pressure, central venous pressure, and cardiac output). These measurements provide local magnitude information but do not capture spatial organization or material history of blood transport. Panel (**B**) depicts the proposed Lagrangian framework, in which blood elements are tracked through space and time to characterize pathlines, streaklines, and Lagrangian coherent structures (LCS). This approach may enable spatial–temporal integration of flow dynamics, reconstruction of material transport pathways, and quantification of distributed energy dissipation across the cardiovascular system.

**Figure 3:**
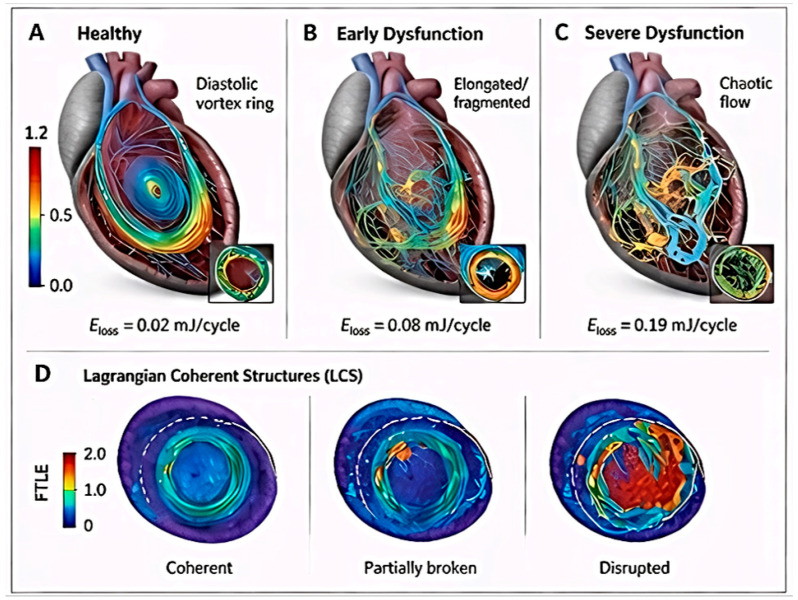
Conceptual demonstration of Lagrangian coherent structures and ventricular flow efficiency across stages of cardiac dysfunction. Panel (**A**) shows normal ventricular filling characterized by a stable diastolic vortex ring that preserves coherent flow organization and minimizes viscous energy loss (E_loss_). Panel (**B**) illustrates early dysfunction, in which the vortex becomes elongated and fragmented, reflecting partial disruption of flow coherence and increased energy dissipation. Panel (**C**) demonstrates severe dysfunction with chaotic intraventricular flow and substantial loss of organized transport efficiency. Energy loss per cardiac cycle increases progressively across stages (healthy: 0.02 mJ/cycle; early dysfunction: 0.08 mJ/cycle; severe dysfunction: 0.19 mJ/cycle). Panel (**D**) displays FTLE maps identifying Lagrangian coherent structures. Coherent rings indicate stable transport barriers, whereas fragmented or disrupted structures indicate breakdown of organized flow topology.

Traditional hemodynamic analysis relies on Eulerian measurements (pressure and flow at a fixed point) that incompletely describe the dynamic energy of the system. In contrast, LCS define the material history and organization of the fluid itself, providing a fundamental description of blood flow topology and its efficiency (Figure 2). LCS are the dominant material curves and surfaces (like moving separatrices) that organize blood flow, particularly within the ventricles and great vessels. A key feature in the left ventricle is the formation of a stable diastolic vortex ring [19], which ensures efficient kinetic energy transfer from the left ventricular (LV) inflow to the outflow tract during systole. The stability of this vortex minimizes viscous energy losses. The fragmentation, elongation, or destabilization of this vortex represents a loss of flow coherence and an increase in viscous energy dissipation, which is a potential marker of early ventricular dysfunction (Figure 3). This breakdown could potentially be inferred at the bedside by analyzing the subtle morphology of the arterial waveform (e.g., an increasingly “noisy” or “damped” upstroke) or via color Doppler echocardiography as an increasingly turbulent or disorganized inflow pattern, even when global indices like Ejection Fraction remain preserved. Quantifying the stability and energy efficiency of LCS may provide a conceptual surrogate measure of the heart’s pumping efficiency, independent of preload or afterload changes. Table 2 translates complex topological flow features (LCS, vortex ring status) into their physiological meaning, providing practical “bedside analogues” and methods for clinical inference.

### 3.2. Topological Data Analysis and Hemodynamic Coherence

**Figure 4:**
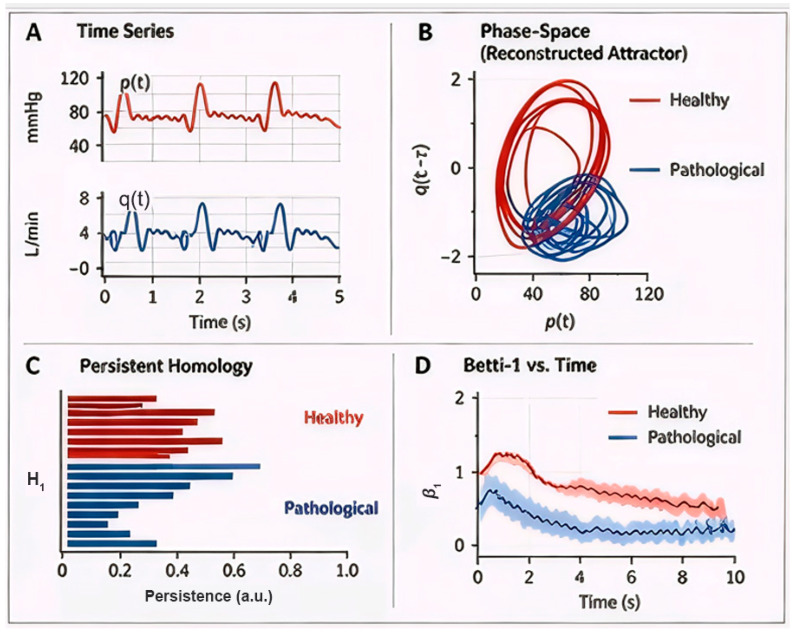
Conceptual topological data analysis of hemodynamic signals and detection of structural coherence loss. Panel (**A**) shows representative arterial pressure and flow time series used for topological analysis. Panel (**B**) presents reconstructed phase-space attractors derived from pressure–flow dynamics. Healthy signals form organized, repeatable trajectories, whereas pathological signals demonstrate irregular, dispersed attractors consistent with reduced dynamical stability. Panel (**C**) displays persistent homology barcodes representing the lifespan of topological features across scales. Longer persistence indicates stable structural organization of the signal. Panel (**D**) shows temporal evolution of the first Betti number (β_1_), representing the number of loops in the reconstructed state space. Reduced β_1_ values indicate collapse of signal complexity and loss of hemodynamic coherence.

TDA provides a powerful, coordinate-free mathematical approach to quantify the “shape” or structural organization of complex hemodynamic time series, such as high-frequency pressure-flow data (Figure 4) [20]. TDA uses techniques like persistent homology to detect and quantify structures (e.g., loops, voids, or “holes” in the data space) that persist across varying observational scales. For circulatory dynamics, this allows us to define and measure hemodynamic coherence—the structural and temporal organization of the pressure-flow relationship.

A high degree of coherence indicates an efficient and predictable system. A major loss of topological coherence may occur when the complexity of the signal collapses, reflecting a fundamental loss of system coherence (i.e., macro-microcirculatory decoupling). This means the microcirculation may no longer be responding in a synchronized or coordinated manner to macrocirculatory pressure changes. Clinically, this phenomenon can be approximated as a significant decorrelation between simultaneously measured indices (e.g., a poor relationship between pulse pressure and stroke volume), leading to a “fuzzy” or disorganized Doppler spectrum. TDA thus offers a mathematically rigorous quantification of the degree of structural breakdown in the system, potentially contributing to earlier physiological recognition of circulatory decompensation before overt failure. Table 3 focuses on how the “shape” of flow (TDA signature) correlates with energetic efficiency (coherent flow, low energy loss) and how this is manifested in measurable clinical waveforms.

### 3.3. Physics-Informed Neural Networks for Inverse Modeling

While the present manuscript employs a deterministic, physiology-based modeling approach to illustrate feasibility, a natural next step in the evolution of this framework is the integration of physics-informed neural networks (PINNs) [21,22]. PINNs represent a class of neural networks that incorporate known physical laws—expressed as governing differential equations such as the Navier–Stokes equations for fluid flow and Womersley–Fung models for vascular mechanics—directly into the network architecture and loss function [23]. By embedding mechanistic constraints into the learning process, PINNs are well-suited to solving highly non-linear inverse problems, particularly when only sparse and noisy clinical data are available.

In the current work, we do not implement, train, validate, or prospectively evaluate a PINN-based architecture. Instead, we demonstrate the conceptual and computational feasibility of inverse modeling using deterministic, physiology-grounded simulations. However, future development of this framework could leverage PINNs to infer time-varying, spatially heterogeneous parameters that are not directly measurable at the bedside. These may include localized pressure gradients, instantaneous viscous dissipation (as a metric of flow inefficiency), and dynamic estimates of Csys.

Within such an extended architecture, sparse bedside inputs—such as the arterial line waveform, CVP, and echocardiography-derived flow velocities—could serve as constraints for the network. The physics-informed structure would regularize inference, enabling stable reconstruction of high-resolution hemodynamic fields from limited observations. This would, in turn, allow real-time estimation of advanced LCS and TDA metrics within a mechanistically consistent framework.

Accordingly, PINNs should be viewed as a forward-looking implementation strategy that may enhance robustness, scalability, and real-time applicability of the proposed multi-scale hemodynamic framework, rather than as a component of the present conceptual framework.

### 3.4. Pulsatile Hemodynamics and Wave Reflection Phenomenology

The arterial system acts as a transmission line where pressure waves propagate and reflect. The interaction between the forward-traveling wave (generated by ventricular ejection) and the backward-traveling wave (reflected from sites of impedance mismatch) determines the pulsatile afterload. In healthy physiology, wave reflection is timed to return during diastole (type B beat), thereby augmenting coronary perfusion pressure without impeding systolic ejection [24].

**Figure 5:**
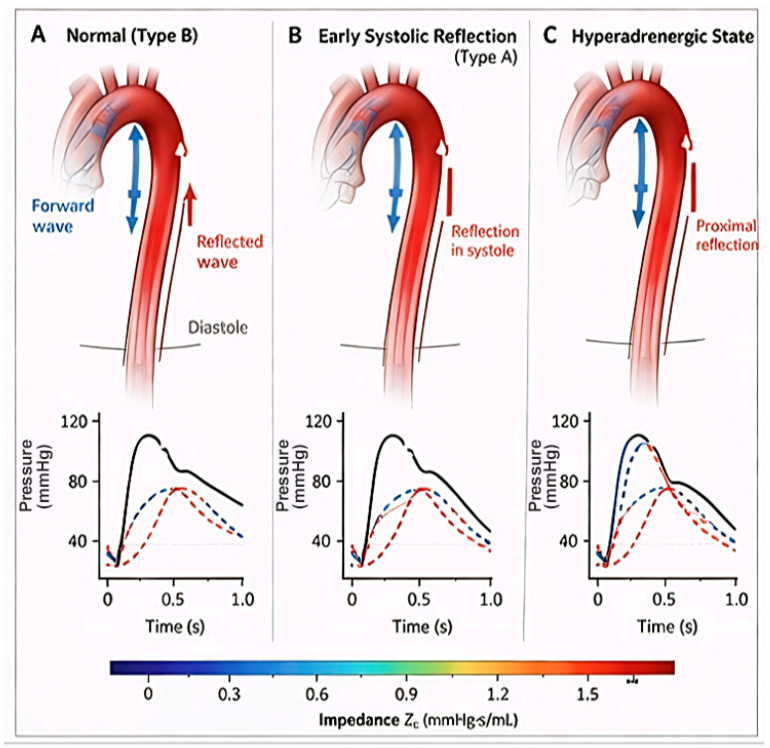
Conceptual demonstration of wave reflection timing and pulsatile hemodynamic phenotypes. Panel (**A**) illustrates normal arterial wave reflection (Type B pattern), in which reflected waves return during diastole and augment coronary perfusion without increasing systolic afterload. Panel (**B**) shows early systolic reflection (Type A pattern), resulting from increased arterial stiffness or impedance mismatch, causing reflected waves to return during systole and increase left ventricular afterload. Panel (**C**) depicts a hyperadrenergic state characterized by proximal reflection and exaggerated systolic pressure augmentation due to widespread arterial stiffening. Pressure waveforms demonstrate the interaction between forward and reflected waves across phenotypes. The color scale represents characteristic impedance (Zc), reflecting arterial stiffness and wave transmission properties.

Rare hemodynamic states frequently involve a “phase-mismatch” of these reflections (Figure 5). In high-output septic shock, one might expect reduced reflections due to vasodilation; however, the administration of high-dose catecholamines can stiffen the central aorta (increasing the elastic modulus) while the periphery remains vasoplegic. This creates a specific impedance mismatch that causes early systolic reflection (type A beat), paradoxically increasing LV afterload despite a low systemic vascular resistance (SVR) [24,25]. Conversely, in traumatic sympathetic storm, the massive surge in endogenous catecholamines creates a generalized arterial stiffening. This shifts the reflection coefficient to unity and the reflection site proximally, causing a “super-systolic” pressure augmentation that may mask true hypovolemia by maintaining MAP despite a critically low stroke volume.

A structured computational workflow summarizing the proposed data flow architecture, mechanistic state estimation process, and phenotype classification pipeline is provided in the Supplemental File.

## 4. Guytonian Circulatory Physiology

The complexity of flow topology is ultimately regulated by the boundary conditions established by the venous circulation. The Guytonian model is foundational, dictating that venous return drives CO through the pressure gradient between Pmcf and RAP: CO = VR = (Pmcf-RAP)/RVR, where RVR denotes resistance to venous return [26].

### 4.1. Volume Conditions of the Cardiovascular System

The central concept here is the functional partitioning of the total blood volume (Vtotal). The classical model divides into unstressed volume (Vu) and Vs: Vtotal = Vu + Vs. The Vs is the volume when the transmural pressure (i.e., the difference between the intramural and extramural pressure) is above zero. Only Vs stretches the vessel walls beyond their relaxed state, generating the potential energy (Pmcf) required to drive flow back to the heart. In contrast, Vu, residing in the compliant, highly collapsible venules, is hemodynamically inert. The normal ratio of Vu to Vs ranges from 30% to 70% [27].

The transition between Vs and Vu is governed by Csys, which is highly regulated by sympathetic tone. Stressed volume failure is defined as a pathological state where a massive increase in Csys—as seen in deep vasoplegia (sepsis, anaphylaxis) or neurogenic shock—causes a significant internal translocation of volume from the Vs compartment into the expanding Vu reservoir [27]. The patient is normovolemic in mass balance but experiences functional hypovolemia; the Pmcf plummets towards the RAP, the venous return gradient collapses, and flow ceases.

This state is fundamentally different from true hypovolemia (total mass loss). In Vs failure, fluid resuscitation simply expands the already excessive Vu reservoir, leading to edema and organ congestion without substantially raising Pmcf [28]. The definitive therapy is the use of α1-adrenergic agonists (vasopressors) to decrease Csys, thereby actively recruiting volume from Vu back into the Vs compartment, restoring the Pmcf/RAP gradient.

### 4.2. Rest Volume

Some recent physiological and theoretical investigations have proposed the existence of a third functional volume component, termed rest volume (Vr) [29,30], although this construct remains incompletely validated. Unlike other intravascular compartments, Vr cannot be recruited or transformed into Vs without an external intervention that either raises vascular tone through vasopressors or lowers arterial and venous resistance, for example, by reducing the dose of pure α-adrenergic agents such as phenylephrine. This unique behavior makes Vr an especially intriguing and clinically relevant physiological construct. Brengelmann previously used a similar term to describe the upper portion of the Vu, beyond which added fluid begins to stretch vascular walls and generate Vs [31]. However, Vr and Vu are not interchangeable, even though both exist near a transmural pressure of approximately zero. Under normal circumstances, Vu can be mobilized when circulatory conditions require it, whereas Vr appears fundamentally inaccessible without an external shift in vascular tone.

Within this exploratory framework, Vr has been hypothesized to serve two potential functions at steady state. First, it helps prevent an increase in venous resistance, and second, it contributes to the maintenance of critical closing pressure—the threshold pressure below which small vessels collapse and capillary perfusion ceases. Because critical closing pressure depends on vascular smooth muscle tone, Vr may contribute to the peripheral venous pressure required to sustain vasomotor reflexes that preserve this threshold [32,33]. Evidence from prolonged septic shock supports this concept: profound arterial hypotension has been associated with marked elevations in venous resistance, particularly within distal segments of the splanchnic circulation [27,34,35]. These observations raise the hypothesis that preservation of Vr-related volume conditions may influence microcirculatory integrity, and that aggressive or inappropriate manipulation of vascular tone may disrupt this volume in ways that impair microcirculatory perfusion. This mechanism may underlie the harmful effects seen when high doses of adrenergic agonists are administered, especially in hypovolemic states.

In severe septic shock, even when vasopressors are administered, the markedly expanded Vu may not be fully reconverted to Vs. As a result, Vr may increase (reflecting the portion of Vu that cannot be recruited) while the Vtotal decreases. Under such circumstances, escalating vasopressor doses increases arterial resistance, impedes blood flow leaving the arterial system and worsens capillary perfusion [27,30,36]. Clinically, this scenario may represent the point along the septic shock continuum at which fluid administration becomes beneficial by restoring Vs, improving CO and enhancing tissue perfusion. The aforementioned observations place Vr as a potentially important factor in maintaining microcirculatory integrity, even at low systemic pressures. Nevertheless, Vr should presently be considered an exploratory physiological construct rather than an established component of the volume conditions of the cardiovascular system.

## 5. Heart–Lung Interaction

The pulmonary circulation serves as a low-pressure, high-compliance coupler between the right and left heart. Its impedance characteristics are uniquely susceptible to changes in intrathoracic pressure and lung volume, creating complex feedback loops during mechanical ventilation.

### 5.1. West Zones and Dynamic Resistor Starling

Pulmonary blood flow is governed by the classic West zone model, which depends on the relationship between alveolar pressure, pulmonary arterial pressure, and pulmonary venous pressure. In rare forms of ventilator-induced cor pulmonale, the application of excessive PEEP can shift lung regions normally operating in Zone 3 (continuous flow) into Zone 1 (no flow) or Zone 2 (waterfall flow). The same phenomenon may occur during careless mechanical ventilation in patients with severe circulatory shock, as well as during ventilation with very large tidal volumes in the course of cardiopulmonary resuscitation. These transitions markedly increase PVR by compressing alveolar capillaries. At the same time, overdistension of the lungs stretches extra-alveolar vessels, producing the characteristic U-shaped relationship between PVR and lung volume [37]. The result is the formation of a functional “hydraulic choke point” at the RV outflow, which can severely compromise RV performance.

### 5.2. Ventricular Interdependence

The heart operates within the rigid pericardial sac, meaning the LV and RV compete for space. In states of acute RV pressure overload (e.g., massive pulmonary embolism or aggressive ventilation), the RV dilates and the interventricular septum shifts leftward (the “D-shaped” septum). This phenomenon, termed ventricular interdependence or direct ventricular interaction, reduces LV end-diastolic compliance [11]. Clinically, this mimics hypovolemia (low LV volumes), yet the administration of fluid further distends the RV, worsens septal shift, and precipitates a spiral of low output. This maladaptive transition represents a progression to the descending limb of the ventricular function (Starling) curve, where increases in preload paradoxically worsen cardiac performance and propagate hemodynamic collapse [11].

## 6. Clinical Expression of Rare Hemodynamic Phenotypes in Shock, Trauma, and Critical Illness

Whereas Section 2 focused on the mechanistic and physiological basis of these phenotypes, the present section emphasizes their bedside clinical expression, dynamic behavior during physiological stress, diagnostic clues, and practical implications for management in critically ill patients. Rather than revisiting the underlying pathophysiology, the emphasis here is on how these states manifest clinically, evolve during monitoring or therapeutic interventions, and influence bedside decision-making.

### 6.1. Macro–Micro Circulatory Decoupling

At the bedside, macro–microcirculatory decoupling is characterized by persistent evidence of tissue hypoperfusion despite apparently acceptable systemic hemodynamic variables. Patients may demonstrate an acceptable MAP or even a seemingly adequate CO, yet remain visibly underperfused. Capillary refill is often prolonged, mottling persists, lactate levels fail to clear despite restoration of global pressure targets, and peripheral perfusion signals remain flat or poorly pulsatile [4]. Sublingual microcirculatory imaging, when available, reveals heterogeneous or disorganized flow despite adequate upstream parameters [26].

Clinically, this phenotype often becomes apparent when progressive escalation of vasopressor therapy fails to improve tissue perfusion and may even worsen peripheral circulation by disproportionately increasing proximal arterial pressures without restoring microvascular recruitment or shear-mediated vasodilation [4]. Concurrent edema-related increases in diffusion distance may further impair oxygen delivery at the tissue level, creating a state in which systemic parameters appear stable while cellular perfusion deteriorates. Recognition of this pattern underscores the limitations of relying exclusively on macrocirculatory targets and supports concurrent assessment of peripheral perfusion, lactate kinetics, and microcirculatory markers when feasible. Management considerations include cautious titration of vasoactive agents, avoidance of unnecessary vasoconstrictive escalation, and strategies aimed at reducing edema burden or preserving glycocalyx integrity when appropriate [38].

### 6.2. Oscillatory Shock During Spontaneous Breathing

Oscillatory shock most commonly becomes clinically evident during transitions from controlled mechanical ventilation to spontaneous breathing, particularly during SBTs. Patients may develop marked respiratory-phase fluctuations in arterial pressure, pulse pressure, CO, and systemic perfusion immediately after initiating spontaneous inspiratory effort. These cyclic oscillations are often accompanied by abrupt hypotension, tachycardia, diaphoresis, or visible respiratory distress and tend to improve rapidly when controlled ventilatory support is restored.

Because these patients may exhibit elevated PPV or SVV, the syndrome may initially resemble classic preload responsiveness. However, unlike true hypovolemia, fluid administration frequently worsens RV loading conditions and amplifies hemodynamic instability rather than stabilizing circulation. Clinicians may therefore observe paradoxical deterioration following fluid boluses together with immediate improvement after reduction in inspiratory effort or restoration of pressure support ventilation.

Recognition of this phenotype is clinically important because management priorities differ substantially from conventional approaches to hypotension during weaning. Therapeutic focus should center on reducing excessive inspiratory effort, optimizing ventilatory support, stabilizing venous return conditions, minimizing abrupt pleural pressure swings, and preventing secondary RV strain. Correct identification of this pattern may prevent inappropriate escalation of fluid administration or vasopressor therapy and instead support individualized ventilatory adjustment strategies.

### 6.3. Pulmonary Vascular Pressure–Flow Dissociation

Pulmonary vascular pressure–flow dissociation manifests clinically as disproportionate hemodynamic sensitivity to relatively small changes in lung volume, airway pressure, or intrathoracic mechanics. In these patients, modest alterations in PEEP, recruitment maneuvers, patient positioning, or spontaneous respiratory effort may precipitate abrupt deterioration in RV performance and systemic perfusion [6]. This behavior is particularly relevant in ARDS, elevated chest wall elastance, pulmonary vascular disease, or preexisting pulmonary hypertension, where regional increases in PVR may rapidly shift RV loading conditions [37].

At the bedside, clinicians may encounter sudden hypotension, new RV dilation or septal shift on echocardiography, abrupt reductions in LV preload, or marked variability in hemodynamic performance after otherwise routine ventilatory adjustments [17]. Conversely, rapid improvements in RV function and systemic perfusion may occur after proning, recruitment optimization, or modest reductions in transpulmonary stress. Clinically, these transitions may resemble hemodynamic bifurcation points in which relatively minor perturbations produce disproportionately large circulatory consequences.

Management therefore requires gradual and carefully monitored ventilatory adjustments with particular attention to avoiding excessive transpulmonary pressure (P_TP_) and abrupt increases in RV afterload. Continuous assessment of RV performance, venous congestion, and tolerance to lung-volume manipulation is essential. Identifying patient-specific ranges of acceptable ventilatory stress may help maintain cardiopulmonary stability in this highly sensitive phenotype.

## 7. Clinical Implementation Challenges

A principal limitation to the immediate clinical deployment of this multi-scale hemodynamic framework lies in the practical constraints of data acquisition and integration in the intensive care environment. Although high-fidelity 4D-flow magnetic resonance imaging and high-resolution computational fluid dynamics provide the reference standard for visualizing Lagrangian coherent structures and quantifying flow topology, these modalities are neither scalable nor feasible in unstable, critically ill patients. Transporting patients in septic, cardiogenic, or oscillatory shock to advanced imaging facilities is frequently contraindicated, and even when feasible, such approaches lack the temporal resolution required for real-time clinical decision support.

The translational bridge for this framework therefore resides not in direct imaging of flow topology, but in solving the hemodynamic inverse problem using routinely available bedside signals. Physics-informed neural networks, or similarly constrained learning architectures, offer a practical implementation strategy. By embedding governing physiological equations—such as the Navier–Stokes relationships for pulsatile flow, Guytonian venous return dynamics, and ventricular–arterial coupling—within the training objective, PINNs allow inference of latent physiological states from sparse, noisy, and partially observed data. Rather than operating as unconstrained predictive models, these systems enforce mechanistic plausibility during learning, reducing overfitting and improving interpretability.

In practice, accessible bedside inputs may include high-frequency arterial pressure waveforms, central venous pressure tracings, ventilator parameters, pulse contour–derived stroke volume estimates, and point-of-care ultrasound Doppler velocities. When temporally synchronized and processed through robust signal quality control—including beat detection, artifact rejection, and respiratory phase segmentation—these measurements can serve as boundary conditions for model inversion. The objective is not to generate purely statistical predictions of hemodynamic status, but to reconstruct hidden state variables such as Vs dynamics, exploratory Vr-related behavior, RVR, pulmonary vascular load sensitivity, and flow coherence metrics that are not directly measurable at the bedside. Vr-related estimates should presently be regarded as exploratory physiological inferences rather than validated measurable cardiovascular compartments.

This strategy may enable the generation of a continuously updated, patient-specific computational representation of the circulation—a physiology-constrained digital twin. Unlike black-box machine learning systems, the inferred parameters correspond to established physiological constructs, preserving interpretability and facilitating clinician trust. The model outputs may include posterior distributions over venous return gradients, right ventricular–pulmonary arterial coupling efficiency, and topology-derived coherence indices, thereby conveying both central estimates and uncertainty. Such probabilistic inference is particularly important in critical care, where measurement noise and rapidly evolving physiology can otherwise lead to overconfident or unsafe recommendations.

Nonetheless, several practical challenges remain. Intensive care unit (ICU) waveform data are frequently affected by damping artifacts, line flushes, arrhythmias, motion artifacts, and inconsistent sampling across devices. Without standardized preprocessing pipelines and vendor-agnostic data harmonization, inference stability may be compromised. Furthermore, the hemodynamic inverse problem is inherently ill-posed; multiple parameter combinations may reproduce similar observable waveforms. Although embedding physiological constraints mitigates this ambiguity, formal identifiability analysis and uncertainty quantification are required to prevent spurious parameter estimation.

Computational latency and integration into bedside workflows also represent non-trivial barriers. For actionable physiology-informed decision-making, inference must occur within seconds to minutes and be seamlessly integrated into existing monitoring systems and electronic health records. This necessitates optimized architectures, edge-computing strategies, and adherence to interoperability and cybersecurity standards. Additionally, explicit safety guardrails must be encoded to prevent physiologically hazardous recommendations, particularly in states involving right ventricular vulnerability or pulmonary vascular instability.

Ultimately, the clinical adoption of this framework depends on rigorous prospective validation. Demonstrating improved detection of rare hemodynamic phenotypes, earlier identification of regime transitions, or safer intervention ranking requires multi-center evaluation with clinically meaningful endpoints. Beyond predictive accuracy, outcome measures should include time to hemodynamic stabilization, reduction in iatrogenic harm, and preservation of organ perfusion.

In summary, the feasibility of implementing multi-scale precision hemodynamics at the bedside does not depend on replicating advanced imaging modalities, but on leveraging physiology-constrained AI to reconstruct latent circulatory structure from routine ICU signals. By transforming sparse measurements into mechanistically interpretable state estimates within a digital twin architecture, this approach offers a pathway toward topology-aware, uncertainty-informed clinical decision support that aligns AI with foundational cardiovascular physiology rather than replacing it.

## 8. Proposed Computational Pipeline for Bedside Physiology-Informed Decision-Making

Accurate hemodynamic assessment in critically ill patients requires continuous interpretation of arterial and central venous pressure waveforms alongside intermittent clinical measurements, yet traditional monitoring largely relies on summary variables such as mean arterial pressure and CO estimates [39,40]. To operationalize the physiologic and topological framework described above, we formalize these elements into a structured computational architecture linking bedside observables to probabilistic phenotype inference and intervention ranking (Supplemental Box). The proposed pipeline transforms routinely acquired ICU signals into multiscale feature representations that integrate waveform morphology, respiratory modulation, and dynamic indices known to reflect cardiopulmonary interaction [41].

The pipeline begins with signal conditioning, beat segmentation, and respiratory phase alignment, followed by extraction of both conventional hemodynamic descriptors and higher-order temporal features. These features are embedded into sliding-window multivariate representations suitable for topological analysis using persistent homology techniques, which have demonstrated robustness for noisy physiologic time series and nonlinear regime detection [42]. This topological layer quantifies coherence and early phase transitions that may precede overt hypotension or shock, thereby extending traditional threshold-based monitoring.

The extracted features then feed a constrained mechanistic inverse layer grounded in established venous return and ventricular–arterial coupling physiology. Rather than inferring state from isolated summary metrics, the architecture estimates posterior distributions over latent physiologic parameters using either deterministic solvers or physics-informed learning strategies that embed governing equations within the inference process [43]. These parameter posteriors are fused with topological coherence metrics and clinical context in a probabilistic phenotype classifier that preserves uncertainty—an approach aligned with contemporary machine learning strategies in critical care physiology-informed decision-making [44,45].

Finally, a counterfactual simulation module evaluates candidate interventions by propagating inferred parameter distributions through the mechanistic core to estimate likely hemodynamic responses and associated risks. Candidate actions are ranked according to expected utility under physiologic constraints, while predefined safety guardrails (e.g., protection against excessive right ventricular afterload during ventilatory adjustment) are enforced [46]. The resulting output is not prescriptive but probabilistic and interpretable, providing structured augmentation of clinician reasoning. In this manner, the proposed pipeline converts physiologically constrained AI into an explicit bedside decision-support system architecture.

## 9. A Case of Advanced Hemodynamic Analysis and Physiology-Informed Therapeutic Reasoning

The patient was a 65-year-old woman with morbid obesity (BMI 52 kg m^−2^), chronic obstructive pulmonary disease with long-term use of supplemental oxygen, pulmonary hypertension (WHO group 3, class 3), coronary artery disease with stent placement, systemic hypertension, insulin-dependent diabetes mellitus, chronic kidney disease (KDIGO G3b), stroke, hypothyroidism, and obstructive sleep apnea. She was admitted to the ICU due to deterioration of her respiratory status (chest x-ray: total atelectasis of the left lung; pH 7.21, PaCO_2_ 130 mmHg, PaO_2_/FiO_2_ 160), thyroid function (thyroid-stimulating hormone 61 μIU mL^−1^) and renal function (KDIGO 3). She was intubated and placed on volume-controlled ventilation with tidal volume 6 mL kg^−1^ p.b.w., PEEP 10 cmH_2_O, and FiO_2_ 0.6. Echocardiography demonstrated preserved left ventricular function, mildly dilated right ventricle with moderate pulmonary hypertension (PASP 45 mmHg), and no major valvular disease. Initial hemodynamics required low-dose norepinephrine (0.05 mcg kg^−1^ min^−1^) to maintain MAP > 65 mmHg.

The patient’s respiratory and renal function gradually improved, thyroid function values normalized, and PEEP was reduced to 8 cmH_2_O with an FiO_2_ of 0.4. Hemoglobin remained 8.4–8.7 g dL^−1^ throughout, with arterial oxygen content (CaO_2_) consistently 11.2–12.2 mL dL^−1^, and attempts at liberation from mechanical ventilation began on ICU Day 10. However, all SBTs resulted in acute hypotension, tachycardia, and hypoxic hypercapnia. Eventually, the patient underwent a tracheostomy due to prolonged mechanical ventilation.

Body composition and clinical status analyses initially revealed a patient with an underlying mechanistic architecture/hemodynamic pattern similar to that of a mixed/alternating “pulmonary” and “extrapulmonary” acute respiratory distress syndrome phenotype: low pulmonary compliance, due to atelectasis (introducing lung stiffness), requiring higher P_TPs_ to open collapsed alveoli and improve oxygenation, combined with a low chest wall compliance, dictating that a large fraction of airway pressure is transmitted to the pleural space, impeding venous return and reducing RV preload. Ventilator settings were individualized by combining driving pressure targets, regional recruitment assessments, moderate permissive hypercapnia, and modest oxygenation targets, which together maintained a ventilatory strategy that the RV could tolerate. However, repeated SBTs were all marked by acute hypotension, variable CO, and unstable oxygen delivery (DO_2_), rapidly followed by severe cardiorespiratory failure. Several other interventions were tested systematically, including fluid therapy modifications and use of norepinephrine/inotropes-inodilators, which temporarily raised MAP and CO but soon worsened pulmonary vascular resistance, aggravating RV loading.

Due to significant logistical hurdles, pulmonary artery and esophageal catheters were not available. Therefore, an integrative methodology, in addition to serial echocardiography, arterial pressure waveform analysis, and tidal volume challenges, was used to deconstruct the predominant hemodynamic phenotype(s) during SBTs with pressure support mode ventilation (PS) and T-piece (Table 4).

**Figure 6:**
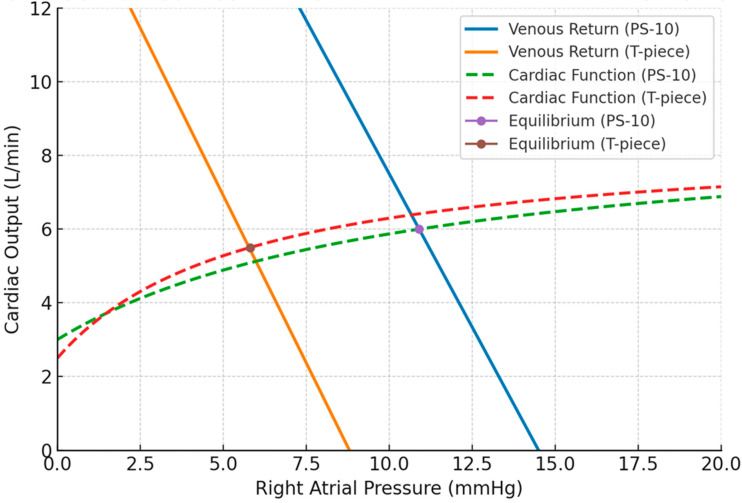
Combined venous return and cardiac function curves. Equilibrium points are shown for PS and T-piece trials, demonstrating a leftward shift in the venous return curve and reduced intersection cardiac output during spontaneous breathing. PS-10, pressure support mode ventilation at 10 cmH_2_O. Curves were constructed from clinical physiological data obtained from the illustrative patient case.

The next SBTs were conducted on PS 10 cmH_2_O/PEEP 5 cmH_2_O. Under these conditions, the patient maintained tidal volumes ~450 mL, a respiratory rate of 20–24 min^−1^, and acceptable gas exchange (PaO_2_ ~85 mmHg on FiO_2_ 0.35). Hemodynamic measurements (see Supplemental File) revealed a Pmcf analog (Pmca) of ~14.5 mmHg, RVR ~1.27 mmHg min^−1^ L^−1^, and CO ~6.0 L min^−1^, with DO_2_ ~900–1000 mL min^−1^. However, transition to a T-piece trial provoked immediate instability, with tachycardia, MAP falling to ~58 mmHg, peripheral oxygen saturation decreasing to ~86%, Pmca decreasing to ~8.8 mmHg, and RVR decreasing to ~0.88 mmHg min^−1^ L^−1^, with CO fluctuating widely (3–8 L min^−1^) and CVP values reaching 0–2 mmHg (Figure 6). The patient exhibited heterogeneous changes in sympathetic tone and selective vascular responses, i.e., preserved arterial constriction and left ventricular–arterial coupling, but absence or weak constriction of the venous network and failure of venous-right heart coupling, followed by severe cardiorespiratory failure within the next 5 min, requiring the re-institution of positive-pressure ventilation.

The aforementioned physiological changes indicated significant variability in preload responsiveness and vascular tone. Central venous pressure was initially stable during PS, falling significantly during T-Piece trials. Dynamic indices such as PPV and stroke volume variation (SVV) changed inversely with CVP, while dynamic arterial elastance (Eadyn) remained stable between PS and T-piece trials. Initiation of norepinephrine resulted in a moderate but temporary decline in PPV and SVV, consistent with improved vascular tone and partial restoration of preload, followed by an increase in PPV and SVV due to an increase in RV afterload.

Due to the complex and atypical cardiorespiratory patterns, we employed simulated weaning strategies based on patient characteristics that included various scenarios of different hemodynamic phenotypes, including venous return physiology, cardiorespiratory interactions, arterial elastance, fluid responsiveness, vasopressor adjustments (norepinephrine vs. AVP), ventilator setting adjustments, inotrope use, and predicted reactions of PPV, SVV, and Eadyn. These simulations provided additional insight. Transition from PS to a T-piece without vasopressors resulted in a clear increase in PPV and SVV, suggesting preload dependence and hemodynamic instability. The addition of norepinephrine during PS-10 improved arterial tone and venous return but increased RV afterload during T-Piece trials. The addition of AVP produced a moderate benefit, while dobutamine conferred only a marginal benefit. The most favorable hemodynamic profile was observed when AVP was used as monotherapy during progressive reduction in PS (10 → 8 → 5 cmH_2_O with FiO_2_ 0.4 → T-piece); this strategy improved venous return and reduced PPV and SVV, normalizing Eadyn without increasing RV afterload, providing the most balanced strategy for successful weaning.

### 9.1. Computational Tools

Due to the unavailability of pulmonary artery and esophageal catheters, an integrative computational methodology was adopted that combined physiologic modeling and conventional monitoring. Advanced signal processing and simulation were performed using Python (Version 3.11) scientific libraries (NumPy, pandas, SciPy). These approaches were employed to deconstruct the predominant hemodynamic phenotypes observed during SBTs and to predict responses to variations in preload, afterload, vasopressor therapy, and ventilator settings.

To further characterize physiologic responses to weaning, seven distinct simulation scenarios were created, each representing clinically relevant combinations of preload, afterload, venous return, vasoactive support, and ventilatory conditions. These scenarios included baseline assisted ventilation, T-piece spontaneous breathing with and without vasoactive support, combined norepinephrine– AVP use, inotrope support with dobutamine, and adjustments in respiratory settings such as reduced respiratory rate and PEEP (Table 5).

The simulated weaning strategies (Table 5 and Table 6) were produced using a deterministic Guytonian mathematical model coded in Python (NumPy/SciPy), which computes venous return, Vs dynamics, Pmca changes, RVR, and ventricular interaction based on established circulatory equations. All numerical outputs—Pmcf, VR, RVR, predicted CO response, and PPV/SVV behavior—were generated from a fixed set of physiologic equations. Thus, the simulations should be considered classical physiology–based rather than PINN-based (physics-informed neural networks) despite the broader theoretical discussion earlier in the manuscript.

**Figure 7:**
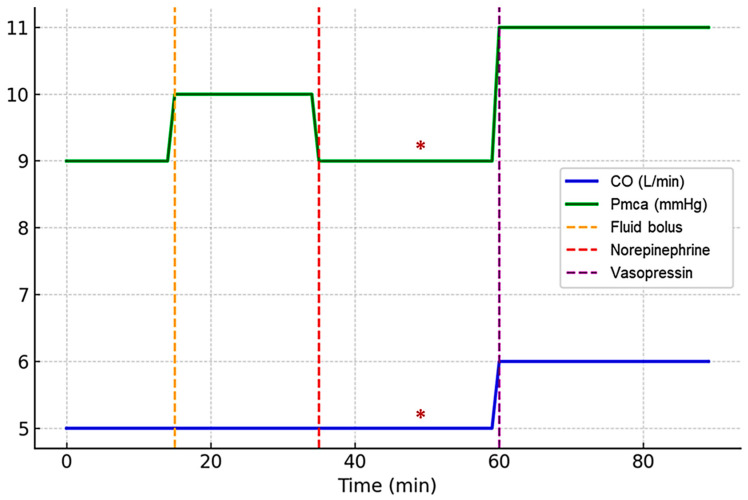
Hemodynamic response to interventions. Vasopressin uniquely stabilized venous return without raising RV afterload, contrasting with norepinephrine and fluids. The star indicates the transition from PS-10 to T-piece. Curves were constructed from clinical physiological data obtained from the illustrative patient case.

Clinical application of the simulated strategy with AVP monotherapy resulted in hemodynamic stabilization (Table 7). An off-label, low-dose AVP (0.01–0.03 U min^−1^) increased Pmca and maintained CO and DO_2_ at 5.5–6.0 L min^−1^ and ~1000 mL min^−1^, respectively (Figure 7), while decreasing PASP. When hypervolemia was evident, it was treated with a continuous furosemide infusion, targeting a CVP < 8 mmHg during PS and <4 mmHg during T-piece trials. In addition, a Boussignac CPAP system was applied three times a day for 2 h to improve oxygenation by opening collapsed alveoli and increasing functional residual capacity. Compared to conventional CPAP systems, the Boussignac device delivers lower actual pressure and airflow [47,48,49,50], preventing excessive P_TPs_ and regional over-distension, especially under high patient demand, which decreases the risk of increased RV afterload and patient-self-inflicted lung injury [12,13,51,52].

**Figure 8:**
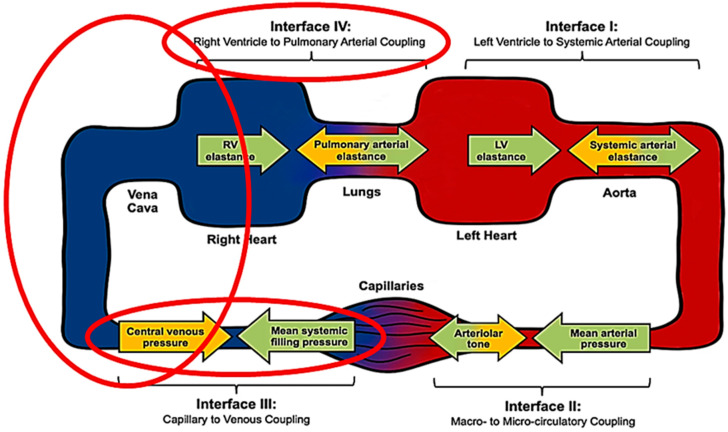
Diagrammatic representation of the main circulatory interfaces. In our patient, failure of the “venous-right heart” and “right ventricle-to-pulmonary artery” circulatory interfaces were evident due to diabetes-induced autonomic nervous system dysfunction. Modified from reference [53] under the terms and conditions of the Creative Commons Attribution (CC BY) license 4.0.

Despite the lack of pulmonary artery and esophageal catheters, our analyses revealed that the most likely scenario was the failure of venous-right heart and right ventricle-pulmonary artery circulatory interfaces due to diabetes-induced autonomic nervous system dysfunction (Figure 8) [53], suggesting AVP as the most appropriate treatment. Eventually, a >30 min T-piece trial succeeded without hemodynamic collapse, and the patient was extubated successfully a few days later.

### 9.2. Structural Geometry of Hemodynamic Failure: A Topological Interpretation of the Case

The central premise of this work is that circulatory failure should be understood not merely as a reduction in flow, but as a disruption in the structural organization of the cardiovascular system. Modern spatial and temporal hemodynamic analysis therefore requires a shift from point-based measurements toward a geometric and topological description of how blood is organized and transported through the circulation. This conceptual transition—from flux to structure—has been emphasized in the framework introduced in Section 3, where Lagrangian coherent structures and related topological constructs are described as the organizing skeleton of cardiovascular flow dynamics.

Within this framework, the case presented here can be interpreted as a transient loss and restoration of circulatory geometry, manifested as a reversible transition between distinct topological states of the venous return system. The observed instability during spontaneous breathing was not primarily a deficit of cardiac contractility or arterial pressure generation, but a geometric reconfiguration of the venous compartment that altered the spatial distribution of Vs and disrupted the structural coherence of venous return.

#### 9.2.1. Geometric Collapse of the Venous Return Manifold

The transition from assisted ventilation to spontaneous breathing produced a rapid and measurable collapse of the venous return driving pressure, defined as the pressure difference between Pmca and RAP (VRdP = Pmca − CVP). This gradient represents the fundamental geometric constraint governing venous return in the Guytonian circulation.

In geometric terms, the circulation can be represented as a dynamic manifold defined by the interaction between pressure, volume, and resistance. Under stable conditions, this manifold maintains a coherent structure that supports efficient energy transfer and stable flow trajectories. During the SBT, however, the system underwent a rapid topological deformation characterized by redistribution of blood from the stressed to the unstressed venous compartment. This redistribution reduced Pmcf and displaced the system away from its stable equilibrium.

**Figure 9:**
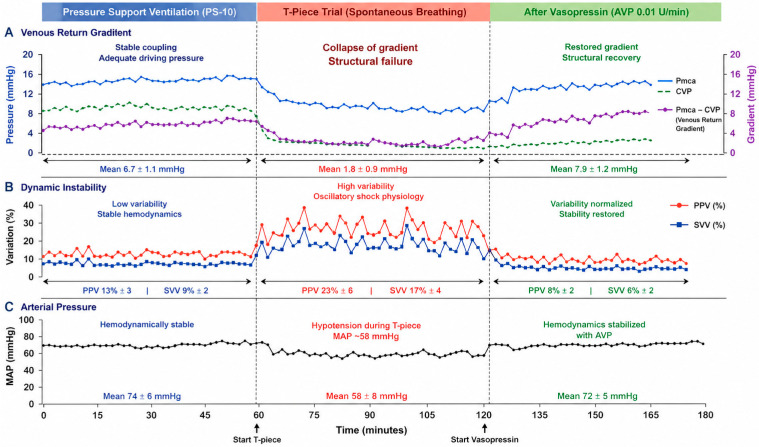
Reversible Structural Failure of Venous Return Coupling During Spontaneous Breathing. Continuous temporal analysis of circulatory structure during transition from pressure support ventilation (PS-10) to spontaneous breathing and subsequent AVP administration demonstrates reversible disruption and restoration of veno-cardiac coupling. Panel (**A**) shows the venous return driving pressure (Pmca − CVP), representing the effective pressure gradient governing systemic venous return, which declines abruptly during the T-piece trial, indicating redistribution of blood from stressed to unstressed venous volume and loss of venous tone. Panel (**B**) depicts dynamic preload indices (pulse pressure variation [PPV] and stroke volume variation [SVV]) that increase markedly during spontaneous breathing, reflecting oscillatory instability and reduced system damping consistent with loss of structural coherence in the cardiopulmonary interaction network. Panel (**C**) demonstrates the associated reduction in mean arterial pressure (MAP), confirming impaired forward flow despite preserved arterial tone. Administration of low-dose AVP restores venous tone through V1 receptor–mediated constriction of venous capacitance vessels, increasing Pmca and re-establishing the venous return gradient, thereby returning the system to a stable attractor state characterized by normalized variability and restored arterial pressure. Collectively, the synchronized changes in gradient magnitude, variability, and perfusion illustrate a reversible structural transition in circulatory organization rather than isolated pump failure, consistent with the principles of modern spatial and temporal hemodynamic analysis. Curves were constructed from clinical physiological data obtained from the illustrative patient case.

This structural transition is directly visualized in Figure 9, where the temporal collapse of the venous return gradient coincides with the emergence of high-amplitude variability in dynamic preload indices and systemic hypotension. The synchronized changes in gradient magnitude, oscillatory variability, and arterial pressure indicate a loss of structural coherence across the cardiopulmonary system rather than a simple reduction in circulating volume.

From a topological perspective, this state can be interpreted as a phase transition in the circulatory system. The stable flow configuration observed during pressure support ventilation corresponds to a coherent attractor state, characterized by low variability and stable coupling between venous return and CO. Initiation of spontaneous breathing introduced large negative intrathoracic pressure oscillations that perturbed this attractor, pushing the system toward a region of instability characterized by fragmented flow trajectories and increased energy dissipation.

#### 9.2.2. Operating Point Migration as a Geometric Trajectory

The hemodynamic evolution of the case can be further interpreted as a trajectory through the phase space of the circulatory system. In this representation, the intersection of the venous return curve and the cardiac function curve defines the system’s operating point, which represents the instantaneous equilibrium between inflow and outflow.

**Figure 10:**
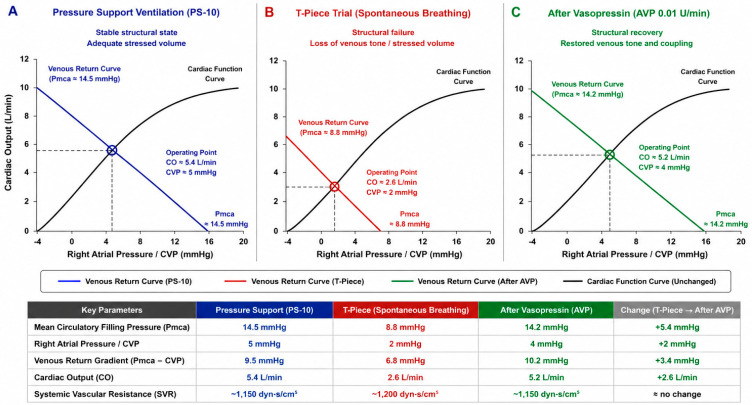
Structural Operating Point Migration in Venous Return Geometry. Geometric representation of circulatory equilibrium demonstrating migration of the operating point across three structural states of the venous return system. Each panel displays the intersection between the venous return curve and the cardiac function curve, defining the instantaneous equilibrium between systemic inflow and cardiac output. Panel (**A**) illustrates the stable configuration during pressure support ventilation, characterized by adequate mean circulatory filling pressure analog (Pmca), preserved venous tone, and efficient forward flow. Panel (**B**) demonstrates structural failure during spontaneous breathing, in which reduced venous tone and redistribution of blood to the unstressed compartment shift the venous return curve leftward, lowering the venous return gradient and displacing the operating point toward a low-output equilibrium despite an unchanged cardiac function curve, indicating that circulatory instability arises from altered venous geometry rather than intrinsic myocardial dysfunction. Panel (**C**) shows structural recovery following AVP administration, where restoration of venous tone shifts the venous return curve rightward, increases stressed volume, and re-establishes a stable operating point without significant change in systemic vascular resistance. This figure conceptualizes hemodynamic instability as a geometric displacement within the circulatory phase space, providing a structural interpretation of reversible circulatory failure. Curves were constructed from clinical physiological data obtained from the illustrative patient case.

Figure 10 illustrates this trajectory explicitly. During assisted ventilation, the operating point resides within a stable region of the phase space characterized by adequate Vs and efficient venous return. Transition to spontaneous breathing shifts the venous return curve leftward, reducing the driving pressure for venous return and displacing the operating point toward a lower-output equilibrium. Importantly, the cardiac function curve remains largely unchanged, indicating that the primary disturbance arises from structural changes in the venous compartment rather than intrinsic myocardial dysfunction.

This migration of the operating point represents a geometric displacement along the circulatory manifold. The system does not fail because the pump becomes weak, but because the spatial configuration of the vascular system changes in a manner that reduces the effective pressure gradient driving flow.

Such geometric interpretation aligns directly with the structural paradigm proposed in Section 3, in which the stability of flow patterns is determined by the organization of the underlying topological structures rather than by isolated scalar measurements.

#### 9.2.3. Efficiency Restoration as Reconstitution of Flow Coherence

The administration of AVP produced a rapid restoration of circulatory stability without substantial changes in systemic vascular resistance. This response indicates that the therapeutic effect was mediated primarily through restoration of venous tone and redistribution of blood volume into the stressed compartment.

**Figure 11:**
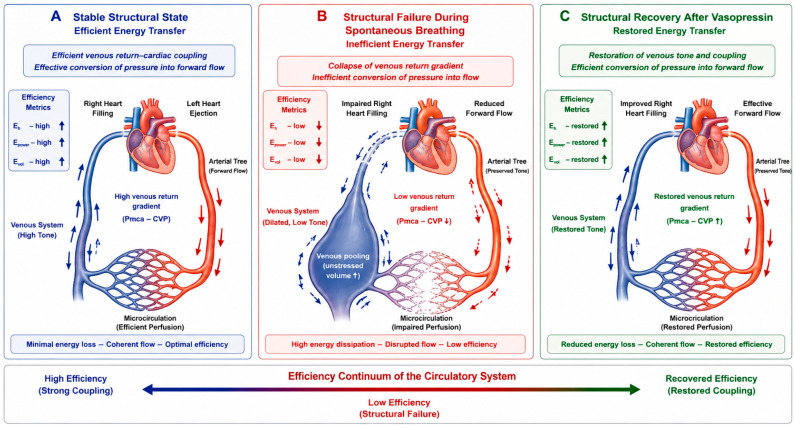
Efficiency-Based Structural Recovery of the Circulatory System. This figure illustrates the energetic consequences of structural transitions in the circulatory system across stable, failure, and recovery states. Panel (**A**) represents the normal configuration in which high venous tone and adequate stressed volume maintain a strong venous return gradient, enabling efficient conversion of pressure energy into forward blood flow with minimal energy dissipation. Panel (**B**) depicts structural failure during spontaneous breathing, characterized by dilation of venous capacitance vessels, redistribution of blood into the unstressed compartment, and collapse of the venous return gradient, resulting in impaired right ventricular filling, reduced cardiac output, and increased energy loss through fragmented and inefficient flow patterns. Panel (**C**) demonstrates structural recovery following AVP administration, in which restoration of venous tone re-establishes the pressure gradient and improves the efficiency of energy transfer across the cardiovascular system. The directional continuum shown at the bottom of the figure represents the transition from a high-efficiency coherent flow regime to a low-efficiency dissipative state and back to a restored coherent configuration. This framework links hemodynamic performance to the structural organization of the circulation and emphasizes efficiency as an emergent property of coordinated vascular and cardiac function. The figure was constructed using physiological data derived from the illustrative patient case and is intended to provide a conceptual representation model.

From a systems perspective, this intervention reconstituted the geometric structure of the circulation, allowing the system to return to a stable attractor state. The restoration of efficient energy transfer is visualized in Figure 11, where normalization of cardiac, power, and volume efficiency indices reflects recovery of coherent flow organization.

Efficiency metrics such as cardiac efficiency (E_h_), power efficiency (E_power_), and volume efficiency (E_vol_) quantify the effectiveness with which the circulation converts pressure energy into forward flow. These variables are defined mathematically as functions of the venous return gradient and CO, providing a direct measure of the system’s energetic performance.

The simultaneous normalization of these indices following AVP administration indicates that the intervention restored the structural integrity of the venous return system rather than simply increasing arterial pressure.

In topological terms, the intervention shifted the system back into a coherent flow regime characterized by stable trajectories and minimal energy dissipation. The disappearance of oscillatory variability and stabilization of arterial pressure therefore represent the re-emergence of a stable flow topology.

#### 9.2.4. Venous Return Geometry as a Structural Biomarker

The geometric representation of the venous return system provides a compact and physiologically meaningful description of the circulatory state. In this framework, the position and slope of the venous return curve encode the structural properties of the venous compartment, including Vs, venous tone, and resistance to venous return.

**Figure 12:**
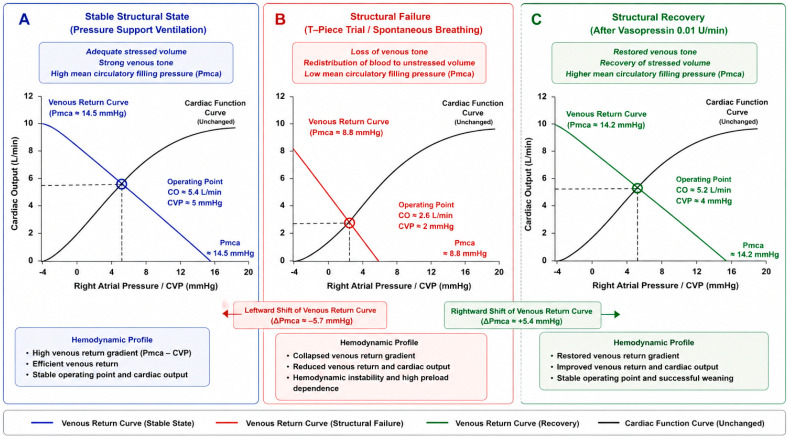
Venous Return Geometry of Reversible Structural Failure. Integrated geometric depiction of reversible structural transitions in venous return dynamics across the clinical course of ventilator liberation. Panel (**A**) illustrates the stable state during pressure support ventilation, where adequately stressed blood volume and preserved venous tone maintain a high venous return gradient and a stable operating point supporting effective cardiac output. Panel (**B**) demonstrates structural failure during spontaneous breathing, characterized by loss of venous tone, redistribution of blood to the unstressed compartment, and a leftward shift in the venous return curve, resulting in reduced cardiac output and hemodynamic instability despite preserved myocardial contractility. Panel (**C**) shows structural recovery following AVP administration, in which venous tone is restored, stressed volume increases, and the venous return curve shifts rightward, re-establishing a stable equilibrium between venous return and cardiac output. The directional arrows quantify the magnitude of geometric displacement in mean circulatory filling pressure analog (ΔPmca), emphasizing that circulatory instability reflects reversible changes in the spatial configuration of the venous system rather than irreversible cardiac dysfunction. This figure synthesizes the structural interpretation of hemodynamic failure as a disorder of venous return geometry within the broader framework of circulatory system dynamics and was constructed using clinical physiological data derived from the illustrative patient case.

Figure 12 demonstrates that the hemodynamic instability observed during spontaneous breathing corresponds to a leftward displacement of the venous return curve, reflecting a reduction in Vs and collapse of the pressure gradient driving flow. Restoration of venous tone shifts the curve rightward, increasing the gradient and re-establishing a stable equilibrium.

This geometric representation provides a structural biomarker of circulatory integrity. Rather than relying solely on pressure or flow measurements, the clinician can assess the spatial configuration of the system and identify early transitions toward instability. Such transitions can be interpreted as topological bifurcations in the circulatory system, where small perturbations in pressure or volume produce disproportionate changes in system behavior. Detection of these bifurcations is central to modern spatial and temporal hemodynamic analysis, which emphasizes the identification of regime transitions rather than the measurement of static variables.

#### 9.2.5. Integration with the Structural Paradigm of Hemodynamic Failure

Taken together, the geometric and topological features observed in this case support the central hypothesis of the present framework: that hemodynamic failure represents a structural disorder of the circulation rather than a simple deficit of flow.

The sequence of events observed in this patient can therefore be interpreted as a reversible structural transition between three distinct states: (i) Stable structural configuration; (ii) Structural failure with loss of flow coherence; and (iii) Structural recovery with restoration of geometric integrity. These transitions are visualized across the temporal, geometric, energetic, and topological domains in Figure 9, Figure 10, Figure 11 and Figure 12. The consistency of these patterns across independent physiological representations provides strong evidence that the observed instability was driven by a coherent structural mechanism rather than by isolated abnormalities in pressure or cardiac function. This structural interpretation aligns directly with the conceptual model introduced in Section 3, in which circulatory stability is determined by the organization and persistence of flow structures within the cardiovascular system. Accordingly, the geometry of venous return and the topology of flow coherence emerge as fundamental descriptors of circulatory function, offering a unifying framework for the diagnosis and management of complex hemodynamic states in critical illness.

### 9.3. Discussion and Clinical Interpretation of the Case

Liberation from mechanical ventilation remains a cornerstone challenge in critical care medicine. While extubation is often framed as a binary event, in reality, it is a prolonged physiologic transition from positive-pressure support to spontaneous negative-pressure breathing. During this shift, a complex set of interactions unfolds between the respiratory and cardiovascular systems. Failure at any link can precipitate weaning failure. Up to 20% of ICU patients experience difficult or prolonged weaning, with mortality rates significantly higher than those who wean successfully [54].

From the perspective of modern spatial and temporal hemodynamic analysis, this transition can also be interpreted as a dynamic reorganization of the circulatory system’s structural geometry. Rather than a simple change in respiratory mechanics, liberation from mechanical ventilation represents a shift in the topology of cardiopulmonary coupling, in which pressure gradients, flow trajectories, and vascular compliance interact to determine system stability. As emphasized in Section 3, the circulation behaves as a structured dynamical system whose stability depends on the persistence of coherent flow patterns and pressure–volume relationships across interacting compartments.

Traditional predictors such as the rapid shallow breathing index, minute ventilation, or gas exchange criteria are limited in scope. They focus primarily on respiratory mechanics and neglect the hemodynamic dimensions of weaning. Yet hemodynamic failure is common during SBTs, manifesting as hypotension, arrhythmias, and/or acute pulmonary edema [17,55]. The physiology of cardiorespiratory interactions is central to this problem. Spontaneous breathing lowers intrathoracic pressure, which increases venous return but also augments RV afterload and alters ventricular interdependence [56,57,58]. In patients with compromised reserve, these changes can trigger collapse.

In structural terms, these events correspond to a deformation of the cardiopulmonary interaction manifold—a geometric representation of the coupled heart–lung system. The stability of this manifold depends on the balance between venous return, cardiac performance, and pulmonary vascular impedance (circuit-heart-lung interaction). When this balance is disrupted, the system may cross a threshold beyond which stable flow trajectories cannot be sustained, resulting in oscillatory instability and hemodynamic collapse. This transition is clearly illustrated in Figure 9, where the synchronized collapse of the VRdP, increased variability in preload indices, and reduction in arterial pressure represent a coordinated loss of circulatory coherence across temporal and spatial domains.

Within the geometric framework introduced in Section 3, the venous return curve can be interpreted as a structural boundary condition governing the circulation. Its slope and position encode the spatial distribution of Vs and the resistance to venous return, thereby defining the feasible region of system equilibrium. Migration of the operating point along this curve represents a trajectory through the circulatory phase space, reflecting the system’s response to physiologic perturbations.

This case highlights the challenges of liberation from mechanical ventilation in patients with complex cardiorespiratory physiology, emphasizing the often-underestimated role of circuit–heart–lung interactions during SBTs. While cardiovascular failure manifests as hypotension, tachycardia, and hypoperfusion, a different underlying physiological mechanism—abolished reflex venoconstriction—can be a primary driver of repeated failure. Traditional indices, such as standard hemodynamic variables or gas exchange, do not capture this dimension and may provide false reassurance in high-risk patients.

Transition from positive-pressure ventilation to negative-pressure breathing significantly decreases intrathoracic pressure. This normally provokes sympathetic activation and triggers reflex venoconstriction to maintain RV preload. However, in our patient, the ANS dysfunction led to a loss of this reflex, causing a precipitous drop in Pmca and venous return. Obesity-related restriction increased the negative pleural pressures generated during SBTs, further exaggerating venous return collapse in the absence of compensatory venoconstriction. The combination of obesity-related chest wall mechanics, pulmonary hypertension, and ANS dysfunction created a perfect storm for preload failure.

From a geometric and topological perspective, this state can be interpreted as a structural bifurcation in the venous return system, in which the redistribution of blood volume altered the configuration of the circulation without directly impairing myocardial contractility. The leftward displacement of the venous return curve during spontaneous breathing, shown in Figure 10, represents this structural transition explicitly. The cardiac function curve remained largely unchanged, indicating that the primary disturbance arose from alterations in venous tone and Vs rather than intrinsic cardiac dysfunction.

Still, the Pmca levels during T-piece trials were maintained at ~8.8 mmHg; this can be explained by the increased intra-abdominal pressure due to morbid obesity and spontaneous breathing, which raised Pmca by promoting blood redistribution from the unstressed to the stressed blood volume. The pressure generated by the arterial system (MAP) also contributed to venous filling and the increasing of Vs.

In our patient, conventional therapeutic strategies proved ineffective. Fluid boluses transiently improved preload but risked exacerbating RV afterload and pulmonary vascular congestion, while norepinephrine restored vascular tone but simultaneously worsened RV loading conditions. Despite the lack of pulmonary artery and esophageal catheters, our analyses revealed that the most likely scenario was the failure of venous-right heart and right ventricle-pulmonary artery circulatory coupling/interfaces due to diabetes-induced autonomic nervous system (ANS) dysfunction [53], suggesting AVP as the most appropriate treatment.

Indeed, low-dose AVP provided a more balanced profile, augmenting Pmca and stabilizing venous return while avoiding increases in pulmonary vascular resistance. This aligns with physiological data showing that autonomic neuropathy in diabetes is associated with orthostatic hypotension, impaired baroreflexes, venous pooling, and poor cardiovascular outcomes [59,60,61]. Importantly, diabetes is prevalent in ICU populations, and up to 20–40% of long-standing diabetics develop significant ANS dysfunction, with impaired baroreflex sensitivity and absent vasoconstrictor responses [59,60]. In such patients, the usual compensatory responses to preload loss during SBTs fail, resulting in hemodynamic collapse (Supplemental Data). Despite its importance, ANS dysfunction is rarely considered in weaning failure algorithms.

The physiologic response to AVP can be interpreted as a restoration of structural coherence within the circulatory system. By increasing venous tone and redistributing blood into the stressed compartment, AVP shifted the venous return curve rightward and re-established a stable equilibrium between inflow and outflow. This restoration of structural integrity is reflected not only in normalized pressure and flow variables but also in improved energetic efficiency of the circulation, as illustrated in Figure 11. The recovery of efficiency indices indicates reconstitution of effective energy transfer across the cardiovascular system, consistent with the re-emergence of coherent flow trajectories described in modern spatial and temporal hemodynamic analysis.

Our findings underscore the relevance of Guyton’s circulatory model, which may aid in deconstructing the interplay between vascular tone, venous return, and CO at the bedside [56,57]. Moreover, this case suggests that AVP may have a role beyond septic shock—as a physiologically rational agent in selected patients with preload collapse and ANS dysfunction during weaning. Future research could explore this hypothesis. Table 2 serves as a reproducibility reference, offering a structured framework of physiologic and therapeutic scenarios that can be replicated or expanded upon in future studies investigating weaning-associated hemodynamic responses.

In critically ill patients with difficult or prolonged weaning, hemodynamic instability—driven by impaired autonomic reflexes and altered venous return physiology—must be considered. From a structural perspective, this instability represents a reversible disorder of circulatory geometry rather than an irreversible failure of cardiac function. The geometric representation of venous return dynamics shown in Figure 12 demonstrates how restoration of venous tone re-establishes the spatial configuration required for stable equilibrium between venous return and CO. Our case, exemplifying a physiological application of the proposed framework, demonstrates that the loss of reflex venoconstriction, compounded by adverse circuit–heart–lung interactions, can lead to recurrent extubation failure. Integrating advanced hemodynamic monitoring, geometric interpretation of circulatory structure, and simulation into clinical practice may enhance precision in managing complex weaning scenarios and ultimately improve patient outcomes.

This illustrative case has several important considerations that should be acknowledged. Invasive reference standards, including pulmonary artery catheterization and esophageal pressure measurements, were not available, limiting direct assessment of cardiopulmonary interactions and ventricular loading conditions. Nevertheless, physiological interpretation and hemodynamic assessment were performed prospectively and in real time at the bedside, reducing the risk of retrospective interpretive bias. In addition, conclusions are derived from a single patient with complex pathophysiology and cannot be generalized to broader critically ill populations. No external validation cohort, comparator methodology, or independent physiological reference standard was available to verify the proposed framework or inferred hemodynamic states. Accordingly, this case should be interpreted solely as an illustrative application of the conceptual framework rather than as confirmatory evidence of diagnostic or predictive validity.

### 9.4. Possible Mechanisms of Autonomic Dysfunction in This Patient

Autonomic dysfunction in this patient likely reflects the convergence of multiple pathophysiologic processes that impair the regulation of venous tone and disrupt the structural integrity of the venous return system. Within the framework of modern spatial and temporal hemodynamic analysis, autonomic control can be interpreted as a dynamic regulator of circulatory geometry, governing the distribution of Vs and Vu blood volume and maintaining the pressure gradient required for stable veno-cardiac coupling. Failure of these regulatory mechanisms produces a loss of structural coherence in the circulation, manifested clinically as instability during physiologic transitions such as SBTs. The mechanisms outlined below represent interacting contributors to this structural vulnerability.

i. Diabetes-related autonomic neuropathy

Long-standing diabetes is strongly associated with damage to small ANS fibers, leading to impaired sympathetic and parasympathetic responses. Pathologic hallmarks include loss of sympathetic ganglionic neurons, baroreflex failure, and denervation hypersensitivity of vascular smooth muscle. Clinically, this manifests as blunted reflex venoconstriction, orthostatic hypotension, and inadequate heart rate/blood pressure responses to stressors—precisely mirroring the loss of reflex venous tone observed during T-piece trials. Mechanistically, hyperglycemia and oxidative stress cause microvascular ischemia of autonomic fibers, accumulation of advanced glycation end-products, and nitrosative injury within sympathetic ganglia.

From a structural perspective, diabetes-related ANS neuropathy impairs the system’s ability to recruit venous capacitance vessels during acute stress, limiting the transition of blood from the unstressed to the stressed compartment. This restriction reduces Pmcf and weakens the VRdP, shifting the circulation toward a low-output configuration analogous to the leftward displacement of the venous return curve shown in Figure 10 and Figure 12. In this context, ANS neuropathy functions as a failure of geometric regulation rather than a primary deficit of myocardial performance.

ii. Critical illness-associated autonomic dysfunction

Prolonged ICU stay, systemic inflammation, and catecholamine exposure can impair central ANS network function (hypothalamic, brainstem, and medullary centers). Persistent inflammation and cytokine release lead to sympathovagal imbalance, characterized by excess baseline sympathetic tone but blunted reflex variability, contributing to paradoxical cardiovascular responses during physiologic transitions such as SBTs. Mechanical ventilation and sedation further dampen baroreceptor sensitivity, compounding the problem.

Within the systems framework of circulatory dynamics, critical illness-associated ANS dysfunction can be interpreted as a disturbance of feedback stability in the central ANS network. Persistent inflammatory signaling alters the responsiveness of baroreflex and chemoreflex pathways, reducing the system’s capacity to maintain coherent flow regulation during rapid physiologic perturbations. The resulting reduction in adaptive variability is reflected in the oscillatory hemodynamic patterns observed during spontaneous breathing, where increased preload indices and unstable arterial pressure indicate a loss of dynamic damping within the cardiopulmonary interaction network, as demonstrated temporally in Figure 9.

iii. Obesity-related autonomic impairment

Morbid obesity alters chemoreceptor and baroreceptor function and reduces vagal modulation of heart rate. Elevated intra-abdominal and thoracic pressures create aberrant mechanoreceptor signaling, which may distort ANS feedback loops during negative-pressure breathing. This can lead to inappropriate sympathetic withdrawal or regional vascular dysregulation, especially in the splanchnic and venous compartments.

In geometric terms, obesity modifies the mechanical boundary conditions of the cardiopulmonary system by increasing intra-abdominal and intrathoracic pressures and altering venous compliance. These changes influence the spatial distribution of blood volume and the transmission of pressure gradients across the thoracoabdominal compartment. During spontaneous breathing, exaggerated negative pleural pressure swings may amplify venous capacitance and promote pooling within the splanchnic circulation, effectively reducing Vs despite preserved total blood volume. This redistribution alters the geometry of venous return and contributes to the structural instability observed during SBTs.

iv. Pulmonary hypertension and right ventricular dysfunction

Chronic RV pressure overload induces altered cardiac afferent signaling and sympathovagal dysregulation. During SBTs, the drop in intrathoracic pressure raises RV afterload; without appropriate sympathetic compensation, RV-pulmonary artery coupling fails, precipitating hypotension and venous collapse. Pulmonary hypertension introduces an additional mechanical constraint on circulatory stability by increasing the energetic cost of forward flow through the pulmonary circulation.

In structural terms, elevated PVR shifts the balance between venous return and CO by increasing the resistance component of the circulatory system. When ANS compensation is impaired, the RV cannot maintain effective coupling with the pulmonary artery, leading to a reduction in forward flow and accumulation of blood within the venous compartment. This interaction amplifies the displacement of the operating point toward a low-output equilibrium, as illustrated geometrically in Figure 10 and Figure 12.

v. Medication and weaning-related neurohumoral imbalances

Chronic catecholamine exposure may reduce the sensitivity of adrenergic receptors, limiting the capacity for rapid venoconstriction during physiologic stress. Simultaneously, withdrawal of positive-pressure ventilation eliminates an external mechanical support that had previously maintained venous return and CO. The transition to spontaneous breathing therefore exposes latent deficits in ANS regulation, allowing structural instability to emerge. This phenomenon is consistent with the abrupt collapse of the venous return gradient observed at the onset of the T-piece trial in Figure 9.

Taken together, the aforementioned mechanisms illustrate that ANS dysfunction in this patient was not attributable to a single cause but rather to the interaction of metabolic, inflammatory, mechanical, and neurohumoral factors that collectively impaired regulation of venous tone. Within the geometry-based framework of circulatory physiology, these disturbances can be understood as failures of structural control over venous return, leading to reduced Vs, diminished pressure gradients, and displacement of the circulatory system toward an unstable equilibrium. This interpretation reinforces the central concept of the proposed framework: that hemodynamic instability in complex critical illness often reflects a disorder of circulatory organization rather than a primary failure of cardiac contractility or circulating volume.

### 9.5. Integrative Mechanistic Summary

The patient’s hemodynamic collapse during spontaneous breathing reflected a failure of the veno-cardiac coupling, which under normal conditions is mediated by rapid sympathetic activation and reflex venoconstriction to preserve Vs. In this case, ANS neuropathy impaired venous recruitment, resulting in a marked decline in Pmcf and venous return despite preserved arterial tone. The coexistence of severe pulmonary hypertension and elevated RV afterload further compromised forward flow, precipitating immediate cardiovascular collapse.

From the perspective of modern spatial and temporal hemodynamic analysis, this event can be interpreted as a transient loss of structural coherence within the venous return system. Under stable conditions, the circulation operates within a coherent geometric configuration in which pressure gradients, vascular compliance, and cardiac performance remain dynamically balanced. During the SBT, this configuration was disrupted by redistribution of blood volume into the venous capacitance compartment, producing a collapse of the venous return gradient and displacement of the system away from its stable equilibrium.

This structural transition is visualized temporally in Figure 9, where the coordinated decline in the VRdP, increased oscillatory variability, and reduction in arterial pressure mark the onset of circulatory instability. In geometric terms, the circulation crossed a stability threshold, entering a regime characterized by fragmented flow trajectories and inefficient energy transfer. Such transitions are consistent with the concept introduced in Section 3 that circulatory stability depends on the persistence of coherent flow structures and pressure–volume relationships across interacting compartments.

The hemodynamic evolution of the case can therefore be understood as a trajectory through the circulatory phase space. The intersection between the venous return curve and the cardiac function curve defines the system’s operating point, representing the equilibrium between inflow and outflow. During spontaneous breathing, reduced venous tone shifted the venous return curve leftward, displacing the operating point toward a lower-output equilibrium while leaving cardiac contractile function largely unchanged. This migration of the operating point is illustrated in Figure 10, confirming that the primary disturbance arose from structural changes in venous return rather than intrinsic myocardial dysfunction.

The coexistence of pulmonary hypertension and elevated RV afterload amplified this instability by increasing the energetic cost of forward flow. In systems terms, the circulation entered a low-efficiency state in which a greater proportion of pressure energy was dissipated rather than converted into effective CO. This inefficient regime is captured conceptually in Figure 11, where reductions in efficiency indices reflect the loss of coherent energy transfer across the cardiovascular system. Restoration of efficient energy conversion following intervention therefore represents not merely symptomatic improvement but reconstitution of the system’s structural integrity.

Through V1 receptor–mediated constriction of venous capacitance vessels, AVP enhanced stressed blood volume independently of adrenergic control and exerted negligible pulmonary vasoconstrictive effects, thereby providing a physiologically coherent strategy to stabilize circulatory dynamics. In geometric terms, AVP shifted the venous return curve rightward by restoring venous tone and increasing the effective pressure gradient driving flow. This shift re-established the spatial configuration required for stable equilibrium between venous return and CO, as depicted in Figure 12.

These observations support a unified mechanistic interpretation of the case: the patient’s instability was the manifestation of a reversible structural disorder of circulatory coupling. The sequence of events can therefore be summarized as a transition between three distinct structural states of the circulation: (1) Stable structural configuration with preserved venous recruitment and efficient energy transfer; (2) Structural failure characterized by loss of venous tone, reduced Vs, and displacement of the operating point; and (3) Structural recovery following targeted restoration of venous tone and re-establishment of circulatory equilibrium. Within the framework of modern spatial and temporal hemodynamic analysis, these transitions can be interpreted as reversible regime shifts in the topology of the circulatory system. Detection of such shifts—through integrated analysis of pressure gradients, flow variability, and geometric relationships—provides a mechanistic basis for physiology-informed hemodynamic management in complex clinical scenarios.

## 10. Conclusions and Future Directions

The integration of modern computational flow analysis, classical cardiovascular physiology, and circulatory structure and coherence presented in this article culminates in a unified framework for precision hemodynamics that is not merely conceptual but computationally operational. By formalizing this framework into an explicit bedside architecture, we move from physiologic interpretation toward structured, reproducible decision support. Within this model, circulatory failure is reframed not solely as insufficient flow magnitude, but as a disturbance in flow organization and circulatory dynamics that may be detected through combined mechanistic inference and topological analysis.

The proposed pipeline demonstrates how routinely available ICU signals can be transformed into probabilistic phenotype inference and counterfactual intervention ranking under physiologic constraints. Importantly, this architecture preserves interpretability through explicit circuit-heart-lung coupling principles while incorporating uncertainty-aware machine learning components. By embedding topology-derived coherence metrics and constrained inverse modeling into a unified system, the approach provides a pathway toward earlier detection of regime transitions that may not be evident through conventional summary variables alone.

Future work should focus on prospective validation of this architecture, including calibration across diverse critical care populations, robustness testing under signal noise and device variability, and evaluation of clinical utility endpoints such as time to hemodynamic stabilization and avoidance of iatrogenic harm. The modular design described in Supplemental Box may enable iterative refinement, including scalable implementation of physics-informed neural networks for real-time parameter inference and expansion toward multimodal data streams (e.g., microcirculatory imaging, advanced Doppler metrics).

The present manuscript proposes a conceptual, physiology-informed framework for interpreting circulatory dysfunction through multiscale structural analysis of hemodynamics. By integrating established cardiopulmonary physiology with emerging computational methodologies, including topology-informed analysis and mechanistic modeling, the framework outlines a translational pathway toward physiologically constrained digital twins capable of continuously assimilating bedside data to estimate latent circulatory states and simulate plausible intervention responses. Unlike purely data-driven prediction systems, such models would aim to preserve mechanistic interpretability and physiological safety constraints grounded in applied cardiovascular and respiratory physiology. Nevertheless, the present framework remains hypothesis-generating and requires substantial computational refinement, prospective physiological validation, and evaluation of clinical utility before any clinical implementation. If successfully validated, this approach could support a transition from predominantly reactive, protocol-driven management toward more predictive, physiology-guided, and topology-aware precision critical care while remaining aligned with the foundational principles of critical care medicine rather than replacing them.

## Figures and Tables

**Table 1:** Mechanistic definitions of under-recognized hemodynamic states.

Hemodynamic State	Core Mechanism	Key Physiological Signature	Bedside Manifestation	How to Detect	Therapeutic Focus
**Stressed volume failure**	↑ Csys → ↓ Vs → collapse of Pmcf–RAP gradient	Loss of effective circulating volume despite normal total blood volume; high Vu, low Vs	Hypotension, low CVP, low Pmcf, poor venous return	Low Pmcf, high Eadyn, minimal response to fluids, blunted sympathetic tone	α-adrenergic vasoconstriction or AVP to decrease capacitance; avoid fluid overload
**Oscillatory shock during spontaneous breathing**	Negative pleural pressure swings → RV afterload sensitivity → cyclic collapse	Phase-locked respiratory instability of RV stroke volume	Large PP variation, pulsus paradoxus, SBT-induced shock	Dynamic arterial waveform swings, Doppler RVOT variation, echo septal shift	Reduce inspiratory effort, apply modest PS/CPAP, treat RV afterload; avoid fluid boluses
**Macro-microcirculatory decoupling**	Microvascular derecruitment + glycocalyx injury despite normalized macrocirculation	Adequate MAP/CO but persistent cellular dysoxia	Mottling, high lactate, low capillary refill, misleading “normal” MAP	Sublingual videomicroscopy, mottling score, veno-arterial CO_2_ gap	Glycocalyx protection, fluid stewardship, vasopressors optimizing microvascular pressure
**Pulmonary vascular pressure–flow dissociation**	Non-linear PVR response to lung volume, recruitment/derecruitment	Abrupt RV loading shifts with small changes in PEEP or position	Sudden RV dilation, hypotension, desaturation during ventilation adjustments	Echo (RV size/strain), changes in PASP, pulse pressure, abrupt instability with ventilator changes	Optimize lung volumes, titrate PEEP, avoid RV overdistension, prone as needed

Csys, systemic venous capacitance; Vs, stressed volume; Pmcf, mean circulatory filling pressure; RAP, right atrial pressure; Vu, unstressed volume; CVP, central venous pressure; Eadyn, dynamic arterial elastance; AVP, arginine vasopressin; RV, right ventricular; PP, pulse pressure; SBT, spontaneous breathing trial; RVOT, right ventricular outflow tract; PS, pressure support mode ventilation; CPAP, continuous positive airway pressure; MAP, mean arterial pressure; CO, cardiac output; CO_2_, carbon dioxide; PVR, pulmonary vascular resistance; PEEP, positive end-expiratory pressure; PASP, pulmonary artery systolic pressure.

**Table 2:** Bridging advanced computational fluid dynamics to bedside monitoring via Lagrangian coherent structures.

Topological Feature	Meaning in Flow	Bedside Analog	How to Infer It
Stable diastolic vortex ring in left ventricle	Efficient energy transfer, low dissipation	Smooth arterial upstroke, narrow PP, stable stroke volume	Arterial waveform morphology; echo color Doppler inflow
Fragmented vortex ring/vortex shedding	↑ Dissipation, RV/LV inefficiency	Noisy upstroke, wide PP, beat-to-beat irregularity	Beat-by-beat variability, rising PPV/SVV, Doppler spectral broadening
Repelling LCS (“transport barriers”)	Regions that prevent mixing → flow stagnation	Regional microvascular shunting	Clinical mottling, lactate, veno-arterial CO_2_ gap
Attracting LCS	Flow convergence zones	Hyperdynamic flow with low perfusion	High CO but poor tissue perfusion markers

PP, pulse pressure; RV, right ventricular; LV, left ventricular; PPV, pulse pressure variation; SVV, stroke volume variation; LCS, Lagrangian Coherent Structures; CO_2_, carbon dioxide; CO, cardiac output.

**Table 3:** Topological data analysis signatures of flow coherence and their clinical waveform analogs.

TDA Signature	Meaning	Clinical Waveform Analog
One long-lived persistent feature	Coherent flow, low energy loss	Quiet, symmetric arterial waveform; stable Doppler envelope
Many short-lived loops	Loss of hemodynamic coherence	“Fuzzy” Doppler spectra, beat instability, variable PP
Rapid topological phase transitions	Approaching bifurcation point	SBT-induced oscillatory shock, sudden RV collapse

TDA, topological data analysis; SBT, spontaneous breathing trial; RV, right ventricular.

**Table 4:** Integrative physiological assessment of hemodynamics.

**1. Venous Return Curves**
Graphs of venous return vs. right atrial pressure
Guyton curves (venous return and cardiac function curves)
Mean systemic filling pressure and the factors that determine it
**2. Cardiac Function Analysis**
Cardiac output curves vs. preload–afterload curves
Frank-Starling relationships
**3. Resistance Calculations**
Venous compartment resistance, resistance to venous return, arterial compartment resistance, systemic vascular resistance
Pressure–flow relationships
**4. Shock Type Analysis (if present)**
Single type of shock
Mixed shock (>1 type)
**5. Waveform Analysis**
Interpretation of arterial waveform
Interpretation of central venous pressure waveform
**6. Simulation with Specialized Hemodynamic Analysis**
Simulation of the effects of fluid administration, vasoconstrictors, and inotropes/inodilators on different cardiovascular/circulatory interfaces
**7. Graphical Representations**
Superimposition of venous return and cardiac function curves
Display of operating points before and after interventions
**8. Final Interventions**

**Table 5:** Simulated weaning scenarios and parameter sets.

	Key Physiologic Assumptions	Vasopressor/Inotrope Setting	Ventilation Mode	Expected Hemodynamic Behavior	Rationale/Clinical Analogy
**Baseline assisted ventilation**	Stable preload and afterload; normal venous tone; Eadyn ~1.2	None	PS-10 (assisted)	Stable CO, low PPV/SVV	Baseline condition before SBT; reflects partially supported patient
**T-piece 100% (spontaneous trial)**	Decreased intrathoracic pressure → ↑ venous return fluctuation; high preload dependence	None	T-P100	PPV ↑, SVV ↑, Eadyn ↓	Simulates SBT failure with excessive negative inspiratory swings
**PS-10 + NE**	Preserved arterial tone, mild increase in venous return pressure gradient	NE 0.05 µg kg^−1^ min^−1^	PS-10	PPV ↓, SVV ↓, MAP ↑	Reproduces clinical scenario with improved tone and perfusion
**T-piece 60% + AVP**	Enhanced venous tone via V1a; arterial tone unchanged	AVP 0.01 IU min^−1^	T-P60	PPV mildly ↓, SVV ↓, Eadyn stable	Simulates partial recovery of preload with venoconstriction
**Combined NE + AVP**	Both arterial and venous tone optimized	NE 0.03 µg kg^−1^ min^−1^ + AVP 0.01 IU min^−1^	T-P60	PPV/SVV lowest, stable CO	Replicates maximal pharmacologic synergy during the weaning phase
**T-piece + DOBU**	↑ Contractility; improved RV output; unchanged preload	Dobutamine 2.5 µg kg^−1^ min^−1^	T-P60	CO↑, PPV/SVV moderate	RV support in pulmonary hypertension
**Low-PEEP/Reduced RR strategy**	↓ Mean airway pressure → Improved venous return, less afterload	None	T-P60 (low RR)	PPV stable, improved preload	Models weaning optimization by ventilator adjustment

Eadyn, dynamic arterial elastance; PS, pressure support mode ventilation; CO, cardiac output; PPV, pulse pressure variation; SVV, stroke volume variation; SBT, spontaneous breathing trial; T-P, T-piece; NE, norepinephrine; AVP, arginine vasopressin; DOBU, dobutamine; RV, right ventricular; PEEP, positive end-expiratory pressure; RR, respiratory rate.

**Table 6:** Evolution of patient physiology after the application of weaning simulations.

Date, Time	Ventilation Mode	PPV (%)	SVV (%)	Eadyn	CVP (mmHg)	Vasoactive Agent
12 June 2025 17:00	PS-10	14	11	1.3	9	None
13 June 2025 01:30	PS-10	18	14	1.5	6	None
13 June 2025 03:00	T-P100	23	19	1.2	6	None
14 June 2025 11:50	PS-10	11	9	1.2	8	Norepinephrine 0.05 mcg kg^−1^ min^−1^
14 June 2025 14:00	T-P60	13	11	1.4	6	AVP 0.01 IU min^−1^
14 June 2025 16:50	T-P60	10	10	1.1	3	AVP 0.01 IU min^−1^
12 June 2025 17:00	T-P60	8	8	0.9	2	AVP 0.01 IU min^−1^

PPV, pulse pressure variation; SVV, stroke volume variation; Eadyn, dynamic arterial elastance; CVP, central venous pressure; PS, pressure-controlled ventilation (at 10 cmH_2_O); T-P, T-piece (at FiO_2_ 1 or 0.6); AVP, arginine vasopressin.

**Table 7:** Simulation of potential weaning strategies.

**1. Key findings during the spontaneous breathing transition**
Major variability in sympathetic tone and selective vascular responses → Significant hemodynamic disturbances, especially venous–cardiac coupling
Reduced sympathetic tone and/or peripheral vasodilation
Reduced effective preload and venous return
Venous compliance (Rven) showed greatest variability, affecting preload and return. Decreased venous tone was key in altering flow dynamics
DO_2_ improvement largely due to better RV-PA, RV-LV, and LV-AO coupling
**2. Recommended treatment strategy**
Low-dose AVP (0.01–0.03 IU min^–1^) during PS → T-piece trials, tapered progressively until extubation, to support venous return and MAP without raising PVR
Add low-dose norepinephrine if sympathetic tone decreases further
Targeting a CVP < 8–10 mmHg during PS and <4 mmHg during T-piece trials
If fluids indicated → administer carefully (100–250 mL over >10–30 min) to allow adaptation and avoid vascular resistance drop
Reassess venous return and RV function after fluid bolus
Avoid abrupt increases in preload or afterload to protect RV
Modified SBT with progressive weaning (PS 10 → 9 → 8 → 7 → 6 → PS 5 with PEEP 5 cmH_2_O and FiO_2_ 0.4 for >60 min, followed by a T-piece), while continuously monitoring hemodynamics to optimize cardiopulmonary interaction
Intensive hemodynamic monitoring—Advanced cardiorespiratory physiology assessments

RV-PA, right ventricular-pulmonary arterial; RV-LV, right ventricular-left ventricular; LV-AO, left ventricular-aorta; AVP, arginine vasopressin; PS, pressure support mode ventilation; MAP, mean arterial pressure; PVR, pulmonary vascular resistance; CVP, central venous pressure; PEEP, positive end-expiratory pressure; FiO_2_, fraction of inspired oxygen; SBT, spontaneous breathing trial.

## Data Availability

The clinical data supporting the findings of this study comprise bedside physiologic waveforms (arterial pressure, central venous pressure when available, electrocardiography), ventilator parameters, intermittent laboratory values, and echocardiographic measurements obtained during routine intensive care monitoring, as detailed in the main manuscript and Supplemental Material. These data are not publicly available due to patient privacy regulations and institutional data protection policies and will not be shared. The computational framework described in this work—including deterministic physiology-based modeling of venous return, stressed volume dynamics, mean circulatory filling pressure analog, resistance to venous return, and simulated weaning scenarios—was implemented in Python (Version 3.11) using standard scientific libraries (NumPy, SciPy, and pandas), as outlined in the proposed computational architecture (Supplemental Box S1). Simulations were generated using a fixed set of established cardiovascular equations within a Guytonian circulatory framework. The custom analysis scripts developed for patient-specific hemodynamic reconstruction and simulation are not publicly available and will not be shared. The manuscript and Supplemental Materials provide a detailed methodological description sufficient to permit independent conceptual replication of the deterministic modeling approach.

## Supplementary Materials

The following supporting information can be downloaded at: https://www.mdpi.com/article/10.3390/jcm15114283/s1, Measurements; Proposed Computational Pipeline for Topology-Based Precision Hemodynamics; and Simulation assumptions and governing equations.

## Abbreviations

The following abbreviations are used in this manuscript:

ABG	arterial blood gas
ABP	arterial blood pressure
ARDS	acute respiratory distress syndrome
AVP	arginine vasopressin
β_1_	first Betti number
Cart	arterial compliance
CO	cardiac output
CO_2_	carbon dioxide
CPO	cardiac power output
CPAP	continuous positive airway pressure
CVP	central venous pressure
Csys	systemic venous capacitance
DAP	diastolic arterial pressure
Ea	effective arterial elastance
Eadyn	dynamic arterial elastance
ECG	electrocardiography
Eh	efficiency of the heart
Epower	power efficiency
Evol	volume efficiency
FTLE	finite-time Lyapunov exponent
LCS	Lagrangian coherent structures
LV	left ventricular/left ventricle
LVOT	left ventricular outflow tract
LVOT VTI	left ventricular outflow tract velocity–time integral
MAP	mean arterial pressure
PASP	pulmonary artery systolic pressure
PE	pulmonary embolism
PEEP	positive end-expiratory pressure
PINN	physics-informed neural network
Pmca	mean circulatory filling pressure analog
Pmcf	mean circulatory filling pressure
PP	pulse pressure
PPV	pulse pressure variation
RAP	right atrial pressure
PS	pressure support
PVR	pulmonary vascular resistance
Q	flow
Rart	arterial resistance
RR	respiratory rate
RV	right ventricular/right ventricle
RVR	resistance to venous return
RVOT	right ventricular outflow tract
SAP	systolic arterial pressure
SBT	spontaneous breathing trial
ScvO_2_	central venous oxygen saturation
SpO_2_	peripheral oxygen saturation
SV	stroke volume
SVR	systemic vascular resistance
SVV	stroke volume variation
TAPSE	tricuspid annular plane systolic excursion
TDA	topological data analysis
TR	tricuspid regurgitation
Vs	stressed volume
VT	tidal volume
Vtotal	total blood volume
VR	venous return
VRdP	driving pressure for venous return
Vr	rest volume
Vu	unstressed volume
Zc	characteristic impedance
