# Supplementary material for: The Geometry of Circulatory Shock: A Conceptual Multi-Scale Lagrangian Framework for Physiology-Informed Hemodynamic Phenotyping
Source: J Clin Med. 2026 Jun 1;15(11):4283. doi: 10.3390/jcm15114283 (PMC13258238; doi:10.3390/jcm15114283)
Supplement: Supplementary file 1 [file jcm-15-04283-s001.zip › jcm-4313147-supplementary.pdf]

SUPPLEMENTAL FILE

The Geometry of Circulatory Shock: A Conceptual Multi-scale Lagrangian Framework for Physiology-Informed Hemodynamic Phenotyping

Authors

Athanasios Chalkias [1,2,3*], Konstantina Katsifa[3], Stavroula Amanetopoulou[3], Georgios Karapiperis[3], Antonios Destounis[3], Ioanna Iatrelli[4], Eleni Laou[5], Athanasios Prekates[3], Paraskevi Tselioti[3]

Departments and Institutions

[1] Institute for Translational Medicine and Therapeutics, University of Pennsylvania Perelman School of Medicine, Philadelphia, PA 19104-5158, USA

[2] OUTCOMES RESEARCH Consortium®, Houston, TX 77030, USA

[3] Department of Critical Care Medicine, General Hospital of Piraeus “Tzaneio”, Piraeus 18536, Greece

[4] Department of Anesthesiology, General Hospital of Piraeus “Tzaneio”, Piraeus 18536, Greece

[5] Department of Anesthesiology, Agia Sophia Children's Hospital, Athens, 11527, Greece

Measurements

Determinants of venous return

The methods of the mean circulatory filling pressure (Pmcf) analogue (Pmca) and related values algorithm have been described in detail before [1-4]. Briefly, based on a Guytonian model of the systemic circulation [CO = $\dot{V}$R = (Pmcf − CVP) / RVR], an analogue of Pmcf can be derived using the mathematical model Pmca = (a × CVP) + (b × MAP) + (c × CO). In this formula, a and b are dimensionless constants (a + b = 1). Assuming a veno-arterial compliance ratio of 24:1, 'a' = 0.96 and 'b' = 0.04, reflecting the contribution of venous and arterial compartments, and 'c' is a combination of veno-arterial compliance ratio (=0.96) and venous compartment resistance (=SVR × 0.038), with the dimensions of resistance, and is based on a formula including age, height, and weight [5,6]:

$$c = \frac{0.96 \times 0.038 \times (94.17 + 0.193 \times [age\ in\ years])}{4.5 \times 0.99^{([age\ in\ years]-15)} \times 0.007184 \times [height\ in\ cm]^{0.725} \times [weight\ in\ kg]^{0.425}}$$

In addition, the following values were determined: Driving pressure for venous return (VRdP) was defined as the pressure difference between Pmca and CVP (VRdP = Pmca – CVP); RVR was defined as the resistance downstream of Pmca to reflect resistance for venous return and was calculated as the ratio of the pressure difference between Pmca and CVP and CO [RVR = (Pmca – CVP) / CO]. This formula is used to describe venous return during transient states of imbalances (Pmca is the average pressure in the circulation and RVR is the resistance encountered to the heart) [7,8].

Efficiency measures

Efficiency of the heart (E_h) was defined as the ratio of the pressure difference between Pmca and CVP and Pmca [E_h = (Pmca – CVP) / Pmca] ($0 \leq E_h \leq 1$). This equation is proposed for the measurement of heart performance, i.e., how well the heart handles the VRdP in terms of Pmca and CVP [1,3,4]. A value ~1 reflects a normal heart function with CVP close to 0. During the cardiac stop ejection, right atrial pressure (i.e., CVP) is equal to the Pmca, and E_h approaches zero [3].

Cardiac power [Power = CO × (MAP – CVP) × 0.0022] and power output [CPO = (CO × MAP) / 451] were also calculated. Cardiac power represents the rate of energy input the systemic vasculature receives from the heart at the level of the aortic root to maintain the perfusion of the vital organs in shock states [9]. Power efficiency (E_{power}) was defined as the ratio between the change in power and the change in Pmca [E_{power} = Δ((MAP-CVP) × CO) × 0.0022 / ΔPmca]. Whereas E_h is a static variable, E_{power} dynamically describes the change in cardiac power in relation to the change in power (MAP × CO) and Pmca [10].

Volume efficiency (E_{vol}) was calculated as the ratio of the pressure difference between Pmca and right atrial pressure (i.e., CVP) and the change in Pmca [E_{vol} = Δ(Pmca-CVP) / ΔPmca] ($0 \leq E_{vol} \leq 1$). Volume efficiency is a dynamic variable embodying the efficiency of added fluid, vasopressor, or inotrope in terms of increasing VRdP in relation to increasing Pmca, and, therefore, CO and oxygen delivery [10].

Other circuit parameters

We also calculated venous compartment resistance [R_{ven} = SVR × 0.038], arterial resistance [R_{art} = MAP / (SV × HR)], arterial compliance [C_{art} = SV / (SAP – DAP)], and effective arterial elastance (E_a = MAP / SV); the latter is an integrative measure of cardiac afterload that includes steady and pulsatile components.

Critical care echocardiography

Transthoracic echocardiography was performed by a highly experienced echocardiographer who was blinded to the individual and study sequence, providing a direct, non-invasive assessment of right ventricular size and function [11]. Pulmonary arterial systolic pressure (PASP) was estimated using the simplified Bernoulli equation with the peak velocity of the tricuspid regurgitation (TR) jet, which provides the pressure gradient between the right ventricle and the right atrium (RA). The estimated right atrial pressure (RAP) is then added to this gradient to find the right ventricular (RV) systolic pressure (RVSP), which equals the PASP in the absence of right ventricular outflow obstruction [12]. The formula is PASP ≈ 4 x $(\text{TR peak velocity})^2$ + RAP (or CVP). All echocardiographic parameters were calculated from five measurements (regardless of the respiratory cycle) and analysed retrospectively. A "fluid challenge," for instance, should ideally be performed under direct echo guidance,

assessing for a positive response (e.g., >15% increase in left ventricular outflow tract velocity time integral (LVOT VTI) while simultaneously ensuring there is no detrimental effect on the right ventricle (i.e., no worsening RV dilation)]. This helps to distinguish patients who are “fluid responsive” from those who are merely “fluid tolerant”.

References

1. Chalkias A, Laou E, Papagiannakis N, et al. Assessment of Dynamic Changes in Stressed Volume and Venous Return during Hyperdynamic Septic Shock. J Pers Med 2022;12:724.
2. Parkin WG, Leaning MS. Therapeutic control of the circulation. J Clin Monit Comput 2008;22:391-400.
3. Chalkias A, Laou E, Mermiri M, et al. Microcirculation-guided treatment improves tissue perfusion and hemodynamic coherence in surgical patients with septic shock. Eur J Trauma Emerg Surg 2022;48:4699-711.
4. Chalkias A, Laou E, Papagiannakis N, et al. Determinants of venous return in steady-state physiology and asphyxia-induced circulatory shock and arrest: an experimental study. Intensive Care Med Exp 2022;10:13.
5. Wijnberge M, Sindhunata DP, Pinsky MR, et al. Estimating mean circulatory filling pressure in clinical practice: a systematic review comparing three bedside methods in the critically ill. Ann Intensive Care 2018;8:73.
6. Moller PW, Parkin WG. Correct calculation of the mean systemic pressure analogue. Intensive Care Med 2022;48:1679-80.
7. Berger D, Moller PW, Takala J. Reply to "Letter to the editor: Why persist in the fallacy that mean systemic pressure drives venous return?". Am J Physiol Heart Circ Physiol 2016;311:H1336-7.
8. Berger D, Moller PW, Weber A, et al. Effect of PEEP, blood volume, and inspiratory hold maneuvers on venous return. Am J Physiol Heart Circ Physiol 2016;311:H794-806.
9. Fincke R, Hochman JS, Lowe AM, et al; SHOCK Investi-gators. Cardiac power is the strongest hemodynamic correlate of mortality in cardiogenic shock: a report from the SHOCK trial registry. J Am Coll Cardiol 2004;44:340-8.
10. Sondergaard S, Larsson JS, Möller PW. The haemodynamic effects of crystalloid and colloid volume resuscitation on primary, derived and efficiency variables in post-CABG patients. Intensive Care Med Exp 2019;7:13.

11. Chalkias A, Katsifa K, Amanetopoulou S, et al. Heart–Lung Interactions in Combined Distributive Shock and ARDS: Applied Cardiopulmonary Physiology at the Bedside. J Clin Med 2025;14:7844.
12. Jang AY, Shin MS. Echocardiographic Screening Methods for Pulmonary Hypertension: A Practical Review. J Cardiovasc Imaging. 2020;28:1-9.

Proposed Computational Pipeline for Topology-Based Precision Hemodynamics (Bedside → Phenotype → Action)

Goal: Operationalize the manuscript's multi-scale framework (Guytonian venous return + heart–lung coupling + flow topology) into a bedside computational pipeline that detects and explains four rarely recognized hemodynamic phenotypes—stressed volume (Vs) failure, oscillatory shock during spontaneous breathing, macro–microcirculatory decoupling, and pulmonary vascular pressure–flow dissociation—and supports intervention selection using mechanistic + topological features.

A. INPUTS

A1. Continuous streams (preferred; near-real time)

Physiologic waveforms (50–250 Hz if available)

- ABP waveform (arterial line)
- ECG (beat timing)
- CVP waveform (if present; important for RAP/CVP behavior)
- Optional: plethysmography waveform (artifact and respiratory modulation support)

Respiratory/ventilator signals

- Mode, RR, VT, inspiratory support, PEEP, driving pressure; timestamps
- Airway pressure/flow waveforms (if available) for breath phase segmentation

A2. Intermittent data (minutes–hours; asynchronous updates)

- Cardiac output (if available), lactate/ABG, hemoglobin
- Vasoactive drug infusion rates (e.g., norepinephrine, vasopressin), fluids/diuresis timestamps
- Echocardiography/Doppler: LVOT VTI (SV proxy), RV size/function (e.g., TAPSE/RV S′), septal shift, Doppler envelope broadening

A3. Context flags

- Sepsis/ARDS/PE suspicion; arrhythmia burden; sedation level; spontaneous breathing trial (SBT) periods

B. OUTPUTS (WHAT THE CLINICIAN RECEIVES)

B1. Primary outputs

1. **Phenotype probability vector** over the four states in Table 1
2. **Mechanistic state estimates (with uncertainty)** consistent with the paper's physiology:

- Vs / venous return gradient behavior (Pmcf → RAP collapse in Vs failure)
- Venous return parameters (e.g., resistance to venous return, RVR; and related constructs used in your Python Guyton model)
- Arterial load indices (e.g., dynamic arterial elastance Eadyn)
- Pulmonary vascular sensitivity to lung volume/PEEP (PVR nonlinearity; RV loading shifts)

3. Ranked candidate interventions with predicted direction/magnitude and confidence:

- e.g., "capacitance restoration (vasopressin / α-adrenergic strategy)", "reduce inspiratory effort / adjust support", "PEEP titration with RV guardrails", "microcirculatory-targeted strategy"

B2. Safety + governance outputs

- Hard-stop alerts (e.g., "RV strain risk: avoid abrupt PEEP increase")
- Data quality report (artifact detection; when not to trust waveform-derived features)
- Explainability summary (top features and physiologic parameters driving the recommendation)
- Audit log (inputs used, versioning, and clinician overrides)

C. COMPUTATIONAL MODULES (END-TO-END)

Module 0 — Time alignment & provenance

- Align all streams to a unified timeline; annotate device sources, sampling rates, calibration/flush events.

Module 1 — Signal quality control (QC), segmentation, and respiratory phase detection

- Beat detection (ECG R-peaks or ABP upstrokes)
- Artifact detection (over/underdamping, flushes, ectopy, motion)
- Respiratory phase segmentation (airway pressure/flow; or ABP respiratory modulation)

Output: QC score $Q(t)$and a "do-not-advise" gate when data are unreliable.

Module 2 — Feature extraction (sliding windows)

Compute robust, windowed features from ABP/CVP/ventilator streams:

- Standard: MAP, PP, HR, systolic upstroke metrics, diastolic decay
- Dynamic/respiratory: PPV/SVV-like surrogates (only when conditions appropriate), inspiratory-expiratory phase contrasts
- Morphology: beat-to-beat instability, "noisy upstroke", notch timing shifts

Module 3 — Flow topology surrogates (LCS-inspired) from bedside analogues

Convert waveform/echo morphology into surrogate markers of flow coherence and inferred vortex/LCS stability, aligned with your "bedside analogues" mapping.

Output: LCS-surrogate indices $L(t)$(e.g., coherence vs fragmentation score).

Module 4 — Topological Data Analysis (TDA) of hemodynamic coherence

- Build sliding-window embeddings of multivariate features (ABP/CVP/respiratory features ± Doppler features when present)
- Compute persistent homology summaries (e.g., number of short-lived loops; dominant persistent feature)
- Detect rapid topological phase transitions (approaching bifurcation points)

Output: topological coherence metrics $T(t)$consistent with Supplemental Table 2.

Module 5 — Mechanistic inverse layer (Guyton-consistent digital-twin core)

Use a constrained inference engine that estimates a posterior over mechanistic parameters θ and latent state x, anchored to:

- Vs/Vu and systemic venous capacitance (Csys) behavior (Vs failure)
- Heart–lung interaction sensitivity (oscillatory shock during SBT)
- Pulmonary vascular nonlinearity (pressure–flow dissociation with PEEP/position changes)

Implementation options:

- Deterministic physiologic solver (as in your current Python Guyton model: Vs dynamics, Pmca/Pmcf behavior, RVR, ventricular interaction)
- Optional PINN wrapper later (same constraints, learned from data; future work described in the manuscript)

Module 6 — Phenotype inference (Bayesian/ensemble classifier)

Fuse:

- TDA coherence metrics $T(t)$
- LCS-surrogate coherence metrics $L(t)$
- Mechanistic posterior summaries $q(\theta, x)$
- Context flags (SBT, ARDS, arrhythmia, vasopressor changes)

Output: $p_k(t) = P(\text{phenotype } k \mid \text{data})$over the four Table-1 states.

Module 7 — Counterfactual simulation & decision support (action ranking)

Generate allowable actions $a \in A$and simulate expected responses (with uncertainty) using the mechanistic core:

- Vs failure: predict effect of capacitance reduction (vasopressin/α-adrenergic), limited fluid responsiveness, Eadyn behavior
- Oscillatory shock: predict effect of reducing inspiratory effort/support adjustments and RV afterload mitigation
- Macro–micro decoupling: de-emphasize MAP-only escalation; prioritize microcirculatory markers/targets
- Pulmonary vascular dissociation: predict sensitivity to small PEEP/position perturbations; RV guardrails

Rank actions by expected utility (benefit − risk) and confidence.

D. PSEUDOCODE (BEDSIDE IMPLEMENTATION)

INPUTS:

Continuous W = {ABP(t), ECG(t), CVP(t)?, Paw(t)?, Flow(t)?}

Ventilator V = {mode, PEEP, VT, RR, driving_pressure, timestamps}

Intermittent H = {CO?, lactate, meds, fluids, echo metrics, context flags}

```
OUTPUTS:
  p(t) = phenotype probabilities over:
        {Vs_failure, Oscillatory_SBT_shock, MacroMicro_decoupling, Pulm_PressureFlow_dissociation}
  q(θ, x | data) = mechanistic posterior (Vs/Csys/Pmcf–RAP behavior; RVR; arterial load; PVR sensitivity)
  R(t) = ranked actions with predicted response distributions + confidence
  Q(t) = data quality report; S(t) = safety flags

ALGORITHM:
0) ALIGN & RESAMPLE:
  T <- unify_timeline(W, V, H)
  W*, V*, H* <- resample_align(W, V, H, T)

1) QC + SEGMENTATION:
  beats <- detect_beats(ECG or ABP, W*)
  resp_phase <- detect_resp_phase(Paw/Flow or ABP_resp_modulation, W*, V*)
  Q(t) <- signal_quality_scores(W*, beats, resp_phase)
  if Q(t) < Q_min:
     return no_recommendation(Q, S=["low_data_quality"])
2) FEATURE EXTRACTION (sliding windows):
  for each window w in sliding_windows(T, L, step=Δ):
     F_std(w)   <- MAP, PP, HR, diastolic_decay, upstroke, CVP_stats
     F_resp(w)  <- resp_phase_contrast(ABP/CVP), PPV_surrogates_if_valid
     F_morph(w) <- beat_to_beat_instability, waveform_noise_metrics
     F_vent(w)  <- PEEP, driving_pressure, mode, inspiratory_support
     F(w) <- concat(F_std, F_resp, F_morph, F_vent)

3) TOPOLOGICAL MODULE (TDA):
  for each window w:
     E(w) <- time_delay_embedding(F(w))
     PD(w) <- persistent_homology(E(w))
```

```
    TDA(w) <- summarize(PD(w))  // coherence vs many short-lived loops; phase transitions
  TopoAlerts <- detect_rapid_transitions(TDA(w))

4) LCS-SURROGATE COHERENCE:
  for each window w:
    L(w) <- lcs_surrogate_from_bedside_analogues(F_morph(w), Doppler_if_present(H*))

5) MECHANISTIC INVERSE LAYER (Guyton-consistent digital twin):
  for each window w:
    θ0 <- initialize_from_features(F(w), context=H*)
    q(θ, x | w) <- infer_mechanistic_posterior(
                  model = venous_return + ventricular_interaction + heart_lung_coupling,
                  init = θ0,
                  observations = F(w),
                  constraints = physiologic_bounds,
                  uncertainty = ensemble/Bayesian)

6) PHENOTYPE INFERENCE:
  for each window w:
    z(w) <- concat(TDA(w), L(w), summary(q(θ, x | w)), context_features(H*))
    p(w) <- Bayesian_or_ensemble_classifier(z(w))

7) COUNTERFACTUAL DECISION SUPPORT:
  A <- generate_candidate_actions(context=H*, phenotype=p(w))
  for each action a in A:
    if violates_hard_safety_rules(a, q(θ, x | w), H*): continue
    Δ(a) <- simulate_response_distribution(mechanistic_model, q(θ, x | w), action=a)
    risk(a) <- compute_risk(Δ(a), RV_guardrails, MAP_floor, arrhythmia_risk)
    score(a) <- expected_utility(Δ(a)) - λ * risk(a)
  R(w) <- rank_by(score, confidence=calibration(q, Q))
```

8) REPORT:

S(w) <- compile_flags(Q(w), TopoAlerts, RV_risk, low_confidence, OOD_checks)

return dashboard(p(w), q(θ,x), R(w), Q(w), S(w), explanations)

E. HARD SAFETY CONSTRAINTS (EXAMPLES ALIGNED WITH YOUR PHENOTYPES)

- **RV guardrails (for oscillatory shock + pulmonary pressure–flow dissociation):**
  If RV dysfunction markers or abrupt instability with PEEP changes are present, block recommendations that increase mean airway pressure abruptly; force stepwise PEEP titration with echo/waveform monitoring.
- **Vs failure guardrails:**
  If inferred Vs failure with minimal fluid responsiveness, penalize repeated fluid boluses and prioritize capacitance-restoring strategy (vasopressin/α-adrenergic) under hemodynamic monitoring.
- **Macro–micro decoupling guardrails:**
  If coherence collapse/decoupling is detected with persistent dysoxia markers, prevent "MAP-only escalation" recommendations unless tissue perfusion markers improve.
- **Data quality guardrails:**
  If ABP damping/flush/ectopy burden high → suppress morphology-driven inference; degrade confidence; show "do-not-advise" if necessary.

Simulation assumptions and governing equations

The simulation framework was constructed as a physiology-informed conceptual model integrating Guytonian venous return physiology, ventricular interaction, arterial load relationships, and respiratory mechanics. Simulations were intended to illustrate mechanistic transitions among hemodynamic phenotypes rather than provide patient-specific predictive estimates.

Guytonian venous return relationships

Cardiac output (CO) was assumed to equal venous return (VR) under steady-state conditions:

$$CO = VR$$

Venous return was modeled according to the Guyton equation:

$$VR = \frac{Pmcf - RAP}{RVR}$$

where Pmcf is mean circulatory filling pressure, RAPis right atrial pressure, and RVRis resistance to venous return.

The mean circulatory filling pressure analogue (Pmca) was estimated as:

$$Pmca = (a \times CVP) + (b \times MAP) + (c \times CO)$$

with:

$$a + b = 1$$

and:

$$a = 0.96, b = 0.04$$

The coefficient c was calculated as:

$$c = \frac{0.96 \times 0.038 \times (94.17 + 0.193 \times age)}{4.5 \times 0.99^{(age-15)} \times 0.007184 \times height^{0.725} \times weight^{0.425}}$$

Driving pressure for venous return was defined as:

$$VRdP = Pmca - CVP$$

Resistance to venous return was calculated as:

$$RVR = \frac{Pmca - CVP}{CO}$$

Volume conditions and compliance assumptions

Total blood volume was partitioned into stressed volume (V_s) and unstressed volume (V_u):

$$V_{total} = V_s + V_u$$

Rest volume (V_r) was modeled conceptually as a non-recruitable component under conditions of impaired vascular tone regulation:

$$V_{total} = V_s + V_u + V_r$$

Mean circulatory filling pressure was assumed proportional to stressed volume and inversely related to systemic venous compliance:

$$Pmcf \propto \frac{V_s}{C_{sys}}$$

where C_{sys} represents systemic venous capacitance.

Transitions from stressed to unstressed volume states were simulated through increases in venous capacitance:

$$V_s \downarrow \text{ as } C_{sys} \uparrow$$

Pressure–flow and arterial load relationships

Effective arterial elastance was defined as:

$$Ea = \frac{MAP}{SV}$$

Arterial compliance was estimated as:

$$C_{art} = \frac{SV}{SAP - DAP}$$

Arterial resistance was calculated as:

$$R_{art} = \frac{MAP}{SV \times HR}$$

Cardiac power was estimated as:

$$Power = CO \times (MAP - CVP) \times 0.0022$$

Cardiac power output was calculated as:

$$CPO = \frac{CO \times MAP}{451}$$

Heart efficiency was defined as:

$$Eh = \frac{Pmca - CVP}{Pmca}$$

Volume efficiency was calculated as:

$$Evol = \frac{\Delta(Pmca - CVP)}{\Delta Pmca}$$

Power efficiency was estimated as:

$$Epower = \frac{\Delta[(MAP - CVP) \times CO] \times 0.0022}{\Delta Pmca}$$

Respiratory interaction assumptions

Respiratory mechanics were modeled conceptually through cyclic pleural pressure fluctuations influencing venous return, right ventricular afterload, and pulmonary vascular resistance.

Transmural pressure was defined as:

$$P_{tm} = P_{intravascular} - P_{pleural}$$

Negative inspiratory pleural pressure swings were assumed to increase venous return transiently:

$$VR \uparrow \text{as} P_{pleural} \downarrow$$

Pulmonary vascular resistance was modeled as a non-linear function of lung volume:

$$PVR \propto f(V_{lung})$$

with a U-shaped pressure–volume relationship characterized by increased pulmonary vascular resistance at both low and high lung volumes.

Right ventricular afterload was assumed to increase during excessive spontaneous inspiratory effort or excessive positive end-expiratory pressure (PEEP):

$$RV\ afterload \uparrow \text{as} PVR \uparrow$$

Topological and structural flow assumptions

Lagrangian coherent structures (LCS) and topological data analysis (TDA) metrics were modeled conceptually as descriptors of flow organization and hemodynamic coherence.

State-space trajectories were reconstructed from pressure–flow time series:

$$x(t) = [p(t), q(t), \dot{p}(t), \dot{q}(t)]$$

Finite-time Lyapunov exponent (FTLE) fields were assumed to identify regions of flow instability and transport separation.

Persistent homology metrics quantified temporal stability of topological features:

$$\beta_0, \beta_1$$

where β_0 represents connected components and β_1 represents loops or cyclic structures in reconstructed hemodynamic state space. Reduced persistence and collapse of β_1 trajectories were interpreted as loss of hemodynamic coherence.

Simulation parameter ranges

Simulations were performed across physiologically plausible ranges representative of shock states and cardiopulmonary instability:

MAP: 40–100 mmHg; CVP/RAP: 0–20 mmHg; CO: 2–10 L min^{-1}; PEEP: 0–20 cmH_2O; SVR: 400–2000 dynes s cm^{-5}; PVR: 50–500 dynes s cm^{-5}; Respiratory rate: 8–40 breaths min^{-1}; Pulse pressure variation (PPV): 5–35%; Stressed volume fraction: 20–40% of total blood volume.

Parameter transitions were modeled dynamically to simulate vasoplegia, right ventricular uncoupling, oscillatory shock, and macro–microcirculatory decoupling states.
